# Whole genome resequencing and comparative genome analysis of three *Puccinia striiformis* f. sp. *tritici* pathotypes prevalent in India

**DOI:** 10.1371/journal.pone.0261697

**Published:** 2022-11-03

**Authors:** Inderjit Singh Yadav, S. C. Bhardwaj, Jaspal Kaur, Deepak Singla, Satinder Kaur, Harmandeep Kaur, Nidhi Rawat, Vijay Kumar Tiwari, Diane Saunders, Cristobal Uauy, Parveen Chhuneja

**Affiliations:** 1 School of Agricultural Biotechnology, Punjab Agricultural University, Ludhiana, India; 2 Regional Station, Indian Institute of Wheat and Barley Research, Flowerdale, Shimla, India; 3 Department of Plant Breeding and Genetics, Punjab Agricultural University, Ludhiana, India; 4 Department of Plant Science and Landscape Architecture, University of Maryland College Park, College Park, Maryland, United States of America; 5 John Innes Centre, Norwich Research Park, Norwich, United Kingdom; Julius Kuhn-Institut, GERMANY

## Abstract

Stripe rust disease of wheat, caused by *Puccinia striiformis* f. sp. *tritici*, (*Pst*) is one of the most serious diseases of wheat worldwide. In India, virulent stripe rust races have been constantly evolving in the North-Western Plains Zone leading to the failure of some of the most widely grown resistant varieties in the region. With the goal of studying the recent evolution of virulent races in this region, we conducted whole-genome re-sequencing of three prevalent Indian *Pst* pathotypes Pst46S119, Pst78S84 and Pst110S119. We assembled 58.62, 58.33 and 55.78 Mb of Pst110S119, Pst46S119 and Pst78S84 genome, respectively and found that pathotypes were highly heterozygous. Comparative phylogenetic analysis indicated the recent evolution of pathotypes Pst110S119 and Pst78S84 from Pst46S119. Pathogenicity-related genes classes (CAZyme, proteases, effectors, and secretome proteins) were identified and found to be under positive selection. Higher rate of gene families expansion were also observed in the three pathotypes. A strong association between the effector genes and transposable elements may be the source of the rapid evolution of these strains. Phylogenetic analysis differentiated the Indian races in this study from other known United States, European, African, and Asian races. Diagnostic markers developed for the identification of three Pst pathotypes will help tracking of yellow rust at farmers field and strategizing resistance gene deployment.

## Introduction

Stripe rust of wheat caused by the fungus *Puccinia striiformis* f. sp. *tritici* (*Pst*) poses a big threat to wheat crop production globally [1, 2]. It can lead to yield losses ranging from 10–100 percent [3] depending upon the resistance level of the varieties under cultivation, prevalence of a matching virulent race, weather conditions, and stage at which infection occurs. In India, North-Western Plains Zone (NWPZ) and the North Hill Zone (NHZ) are more prone to this disease. In these areas, stripe rust has been occurring in moderate to severe form since 2008 and is responsible for causing yield losses as high as 69% [4]. In NWPZ, especially in Punjab, disease symptoms start in second to fourth week of December (i.e., seedling stage) on susceptible varieties due to the existence of favorable microclimate conditions. This early infection acts as a gateway for the secondary inoculum in the form of uredospores for the whole of Punjab and adjoining states. Physiological races of *P*. *striiformis* recorded in India till date are 13, 14, 14A, 19, 20, 20A, 24, 31, 38, 38A, 57, A, G, G-1, I, K, L, M, N, P, Q, T, U, C I, C II, C III and 46S119, 78S8, 110S119 and most recently 238S119 [5]. Cultivation of resistant varieties, timely monitoring of the disease and destruction of the initial infection sites with fungicides is the integrated approach for the management of the disease in hot spot areas and also to prevent its further spread to other areas.

The preferred strategy in terms of economics, environment and farmer safety, is the cultivation of resistant varieties. However, due to the fast-evolving nature of the pathogen, new races of the pathogen evolve with additional virulence, aggressiveness and better adaptability after every 4–5 years [6]. The stepwise evolution of pathotypes is a major threat to breeding stripe rust resistant wheat varieties [7]. Mutations are the natural events in the pathogens and are independent of the host. The acquisition of virulence by the pathogen is a result of the large-scale deployment of a resistant variety, which favours the selection of a virulent mutant of the avirulent pathogen [8]. Such a selection pressure results in new virulent types. A single virulent uredospore can produce billions of identical spores which can spread long distances with the help of wind followed by rapid local adaptation. New races can cause severe epidemics on the previously resistant cultivars [9]. Examples of resistance breakdown include *Yr17* from 1993 to 1999 in Europe and *Yr27* in mega wheat variety PBW343 in India due to the evolution of a new pathotype of *Pst* i.e.78S84 [10]. In the latter case, drastic yield reductions occurred in sub-mountainous areas of Punjab as large area was under PBW343 cultivation which led to severe disease outbreak and hence was responsible for causing 60–80% yield losses [4, 11]. Therefore, monitoring of the pathogen population for virulence changes is essential for the efficient utilization of genetic resistance against stripe rust of wheat.

In Punjab high genetic diversity in *Pst* populations (as identified with SSR markers) and low pathogenic diversity was reported [12, 13]. In recent years, four races of the pathogen namely 46S119, 110S119, 238S119 and 110S84 are present in Punjab and among them, 46S119 and 110S119 are the most prevalent [5, 13]. Whole-genome sequencing approaches can aid in the understanding of the dynamic nature of the *Pst* genome and facilitate the development of rust resistant varieties carrying effective resistance gene(s), which can stay in field over a longer time period. Till date several *P*. *striiformis* pathotypes have been sequenced including PST race 130 [14, 15], PST-21, PST-43, PST-87/7, PST-08/21 [16], isolate12-368 [17], 93–210, 93TX-2 (*P*. *striiformis* f. sp. *hordei*) [18], PST-78 [19], 67S64, 47S102(k), 46S119 [20], *Pst* isolate CY32 [21], *Pst*-104E [22], 11–281 (wheat grass) [23] with varied genome size ranging from 55 to 115 Mb. Kiran *et al*. [20] studied the genomic features and variation among the three *Pst* races occuring in India namely 46S119, 31 and K using whole-genome sequencing. They found that pathotype 46S119 (detected in 1996), most likely emerged through a single-step mutation in pathotype 46S103 leading to loss of its incompatibility to the resistance gene *Yr9*. Aggarwal *et al*., [24] sequenced rDNA-ITS and beta-tubulin in ten *Pst* pathotypes collected from different regions of India and conducted phylogenetic studies with the sequence data of other Asian and US isolates available at NCBI. The sequence similarity of Indian pathotypes with isolates from China and Iran indicated the common origin for Asian isolates. But Asian isolates constitute a distinct evolutionary lineage from US isolates.

In the present study, we conducted whole-genome sequencing of three Indian *Pst* pathotypes Pst46S119, Pst78S84, and Pst110S119. Our aim was to understand the origin of these pathotypes, their relationship with other *Pst* pathotypes sequenced globally, identify pathotype specific virulence genes, and develop markers for monitoring the pathotype profiles in the field.

## Materials and methods

### Materials used and genomic DNA isolation

Three Indian *Pst* pathotypes Pst46S119, Pst78S84 and Pst110S119 with different virulence profiles (Table 1) were multiplied from single-pustules and purified under stringent quality control at Regional station, IIWBR, Flowerdale, Shimla and bulked for DNA extraction. Genomic DNA was extracted from uredospores following the CTAB method [20]. About 50 mg of uredospores were finely ground in liquid nitrogen with the help of a pestle and mortar. Subsequently, 1000μl extraction buffer (Tris-Cl (50 mM), EDTA (100 mM), NaCl (150 mM), and 0.2% β mercaptoethanol at pH 8) were also added to the uredospores powder in a 2.0ml centrifuge tube. The suspension was incubated for 1h at 65°C with gentle shaking at 10-minute interval. Thereafter, the suspension was cooled to room temperature and an equal volume of chloroform: isoamyl alcohol (24:1) was added. The suspension was centrifuged at 12,000 rpm for 10 min at 4°C. The supernatant was transferred to a new centrifuge tube, 0.6 volume of ice-cold isopropanol was added to it and incubated at -20°C for two hours. The mixture was then centrifuged at 12,000 rpm for 10 min at 4°C. The resultant supernatant was discarded, and the DNA pellet was washed with 70% ethanol. The pellets were then dissolved in 500μl triple distilled water and mixed with 500μl of 25:24:1 (saturated phenol: chloroform: isoamyl alcohol) solution. The supernatant was transferred to a new centrifuge tube to which 1/10 portion of Sodium acetate (3M), an equal volume of ice chilled isopropyl alcohol was added and shaken gently. The contents were incubated at -20°C for 2 hours and then centrifuged at 12,000 rpm for 10 minutes at 4°C. The resulting supernatant was discarded, and pellets were washed with 70% ethanol, air dried, and dissolved in 200μl of autoclaved triple distilled water, quantified in NanoDrop 2000R UV-Vis Spectrophotometer (Thermo Scientific Pvt. Ltd, India) and stored at -20°C for further use.

**Table 1:** Avirulence/virulence profile of *Puccinia striiformis* fsp *tritici* pathotypes Pst46S119, Pst110S119 and Pst78S84.

Stripe rust pathotype	Avirulence/Virulence formulae
110S119 = 110E159	*Avirulence*: *Yr1*, *Yr5*, *Yr10*, *Yr13*, *Yr14*, *Yr15*, *Yr16*, *Yr24*, *Yr26*, *YrSp*, *YrSk*
	*Virulence*: *Yr2*, *Yr3*, *Yr4*, *Yr6*, *Yr7*, *Yr8*, *Yr9*, *Yr11*, *Yr12*, *Yr17*, *Yr18*, *Yr19*, *Yr21*, *Yr22*, *Yr23*, *Yr25*, *YrA*, *YrSo*
46S119 = 46E159	*Avirulence*: *Yr1*, *Yr5*, *Yr10*, *Yr11*, *Yr12*, *Yr13*, *Yr14*, *Yr15*, *Yr16*, *Yr24*, *Yr27*, *YrSp*, *YrSD*
	*Virulence*:*Yr2*, *Yr3*, *Yr4*, *Yr6*, *Yr7*, *Yr8*, *Yr9*, *Yr17*, *Yr18*, *Yr19*, *Yr21*, *Yr22*, *Yr23*, *Yr25*, *YrA*, *YrSD*, *YrSo*
78S84 = 78E16	*Avirulence*: *Yr1*, *Yr5*, *Yr10*, *Yr11*, *YrA*, *YrSp*, *Yr14*, *Yr15*, *Yr17*, *Yr18*, *Yr24*, *Yr28*, *Yr29*
	*Virulence*:*Yr2*, *Yr3*,*Yr4*, *Yr6*, *Yr8*, *Yr9*, *Yr12*, *Yr31*, *YrSk*

### Genome sequencing and assembly

Paired-end libraries were prepared for the three pathotypes i.e., Pst110S119, Pst46S119 and Pst78S84 and sequenced using Illumina HiSeq-2500 platform through outsourcing. The quality of raw sequencing reads were assessed using FASTQC version 0.11.5 [25]. Low-quality reads and adapters were filtered-out at Phred quality score of ≥30 with minimum read length of 50bp using Trimmomatic v0.39 [26]. High-quality paired-end reads were further assembled using SPAdes *de novo* assembler [27] and quality of the assembly was assessed by re-aligning filtered reads to their respective isolate assembly. Benchmarking Universal Single-Copy Orthologs (BUSCO v2.0) pipeline was used to evaluate the completeness of the genome assembly based on conservation of single-copy orthologs belonging to Basidiomycota dataset of 1,335 conserved genes [28]. Genome completeness was also accessed by FGMP (Fungal Genome Mapping Project) pipeline [29].

### Repeat annotation, gene prediction and gene family annotation

*De novo* repeat finding tool RepeatModeller, and RepeatClassifier were used to identify genome-specific repeats in the assembled genome of *Pst* pathotypes [30] followed by searching of known repeats in the RepBase database [31]. *De novo* and known repeats were masked using RepeatMasker and masked genome assemblies were further used for gene prediction using GeneMark-ES software [32]. Predicted genes were annotated by blastx search against the non-redundant (NR) database [33] and Blast2GO was used for the assessment of gene ontology (GO) terms and functional assignment [34]. PfamScan program for identification of protein domains [35]. Putative carbohydrate-active enzymes and protease families might be associated with host adaptation were also identified by searching against the dbCAN and MEROPS database [36] [37]. We used the dbCAN-CAZymes classification pipeline to identify CAZymes in Indian *Pst* isolates. Genes involved in Pathogen-Host Interactions were detected through blastp search against Pathogen-Host Interaction database (PHI-base) [38]. Secretory proteins and effectors candidates were predicted using predict_secretome (https://github.com/fmaguire/predict_secretome) pipeline, and machine learning-based tool EffectorP v2.0 software (http://effectorp.csiro.au) which uses SignalP v4.1 [39], TargetP v1.1 [40] and WoLF PSORT v0.2 [41]. Sequences were confirmed for the absence of transmembrane (TM) domain using TMHMM v 2.0c [42, 43].

### Association of pathogenicity related genes with repetitive elements

Association between pathogenicity related genes (CAZyme, proteases, gene involved in pathogen-host interaction, candidate effectors, and secretome proteins) and genomic repeats was performed using permutation tests based approach of regioneR package [44]. regioneR compares the mean distance of the gene from the nearest repeat element against the distribution of distances of random samples in the whole genome. A total of 10,000 random iterations were used for calculating Z-score and associated probability for each gene class in regioneR.

### Ortholog determination and evolution of gene families

Potential orthologs in the proteome of *Puccinia* genera were detected using OrthoFinder [45]. Single copy orthologs were filtered and aligned using MAFFTv.7 [46]. Alignments were trimmed using trimAl v.1.3 [47] to remove positions with the gap in the alignment. Trimmed alignments were concatenated into a single alignment using FASconCAT-G [48]. ProtTest [49] was used to test for the best evolutionary model on the concatenated alignment. RAxML was used to build a maximum-likelihood phylogenetic tree with PROTGAMMAJTT model, and 1000 bootstrap [50]. Gene families were classified via all-vs-all blastp search of eight *Puccinia* species (*i*.*e*. *P*. *triticina* race 1 (BBBD), *P*. *graminis* f. sp. *tritici* (CRL 75-36-700-3), *P*. *striiformis* f. sp. *tritici* (2K41-*Yr9*), *P*. *striiformis* f. sp *tritici* (93–210), *P*. *striiformis* f. sp. *hordei* (93TX-2), and Indian *P*.*striiformis tritici* pathotypes (Pst110S119, Pst46S119 and Pst78S84). Blastp results were clustered into gene families using MCL [51] and family size counts were determined. Species tree generated by OrthoFinder was converted into the ultrametric tree. Computational Analysis of gene Family Evolution (CAFE) [52] was used to identify gene families that undergone significant expansion or contraction in genome. Gene family evolution was also studied for the defined classes of CAZyme, Proteases, Effector, Secretome and pathogenicity related genes.

### Analysis of diversifying selection acting on genes

Selecton program was used to calculate the ka/ks ratio estimate for both positive and purifying selection at each amino-acid site in protein-coding genes [53]. Homologous sequences among the three Indian pathotypes were identified via reciprocal best blast hit at e-value cutoff of 1e^-03^ and aligned using ClustalW [54] After removing the partial genes lacking either the start or stop signal, codon alignment of mRNA sequences was generated using Revtrans [55]. Site-specific Ka/Ks ratio and log-likelihood ratios were calculated using the positive selection model (M8) and null model (M8a) for each gene. Loglikelihood ratio test between M8 and M8a model was performed at one degree of freedom (df). Genes with Ka/Ks ratio >1 at amino acid sites and p-value <0.001 were considered as positively selected genes.

### Mutational analysis

High-quality reads of three *Pst* pathotypes were aligned to their respective assembled genome and also with other two assembled isolates using Bowtie2 [56] and converted into bam files using SAMtools [57]. GATK Haplotypecaller was used for the identification of SNPs from the aligned BAM files [58] and indels were removed using VCFtools [59]. SNPs were filtered at quality score >30 and a minimum read depth of 3 reads. SNP calling was performed for both intra- and inter- isolate samples.

### Phylogenetic analysis

We aligned the assembled contigs of three pathotypes from India with the *P*. *striiformis* whole-genome assembly of strain DK09_11 (Bioproject: PRJNA595755, WGS:WXWX01) using Satsuma [60]. Pseudoscaffolds were developed using OrderBySynteny subprogram of Satsuma and syntenic region were visualized using Circos [61]. Pairwise sequence identity between the pathotypes was calculated using Pyani v:0.2.10 (http://widdowquinn.github.io/pyani/). Whole-genome alignment of eight *Puccinia* genomes, i.e., DK09_11, 2K41-*Yr9*, Pst110S119, Pst46S119, Pst78S84, 46S119, 47S102, and 67S64 was performed using Mugsy [62] considering DK09_11 as reference for alignment. Local phylogenetic relationship and differential segment boundaries were determined using the machine learning-based tool SAGUARO [63].

### Identification of pathotype-specific genes and design of genome-specific markers

Gene prediction (data not shown) was performed in 17 *P*. *striiformis* genomes. Protein orthologs were detected using OrthoFinder in 20 genomes i.e., 17 available *Puccinia striiformis* reference and three assembled Indian pathotypes [45]. Species-specific orthogroup and singletons were identified among the three Indian *Pst* pathotypes. Jellyfish based on Kmer- approach was used for generating Kmers and aligned on other pathotypes using Bowtie2 [56] and finally for designing genome-specific markers [64]. Coverage and position of unmapped Kmers were determined for their respective genome. Continuous mapped regions were used to design primers using Primer3 [65]. NCBI e-PCR was used to detect uniquely mapping genome-specific primers [66]. Forty-five selected gene specific markers were analyzed on different Indian *Pst* isolates along with the mixture of stripe rust inoculum collected from experimental wheat fields and farmer’s field. Primer sequences are given in supplementary **S16 Table in S2 File**. PCR reactions were performed using an Applied Biosciences 96 well thermal cycler in a 12μl PCR reaction mixture containing 2.0 μl template genomic DNA (25 ng/μl), 5.0 μl of 2× EmeraldAmp R GT PCR Master Mix, 2.0 μl of nuclease-free water and 1.5 μl of 5 μM each primer. For PCR amplification, temperature profile comprised initial denaturation at 94°C for 4 min, 40 cycles consisting of denaturation at 94°C for 1 min, annealing at 59°C for 1 min, extension at 72°C for 1 min and a final extension at 72°C for 7 min. The PCR products were resolved on 2.5% agarose gel electrophoresis and visualized using gel documentation system.

## Results

### Whole genome sequencing and *de novo* assembly of Indian *Pst* pathotypes

**Fig 1:**
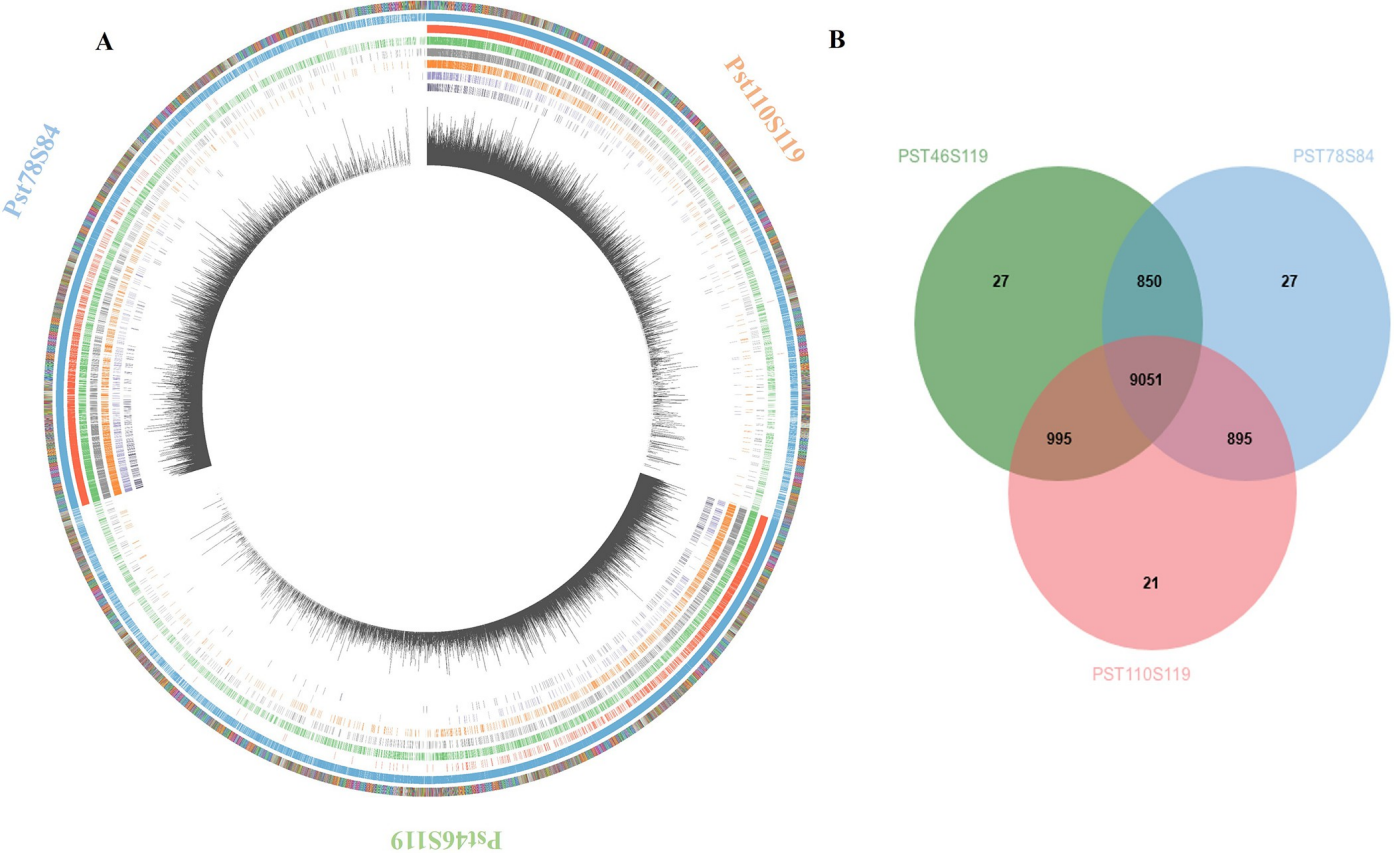
Genome assembly of three *Puccinia striiformis* pathotypes Pst110S119, Pst46S119 and Pst78S84. A) Genome-wide distribution of genes and pathogenicity gene classes of three isolates. Bar in the outer layer denotes one contig, ordered from largest to smallest. Successive layers demonstrate, total number of genes, genes under positive selection, effectors, secreted proteins, pathogenicity homologs from phi-base, proteases, CAZymes and SNP density per kb in three isolates. B) Number of the shared and distinct orthologous group between three pathotypes.

Three *P*. *striiformis* pathotypes from India (Pst110S119, Pst46S119 and Pst78S84) with differential virulence profiles were selected and sequenced using paired-end sequencing on Illumina HiSeq. Raw data of 20.00, 10.69 and 10.29 million reads were generated for Pst110S119, Pst46S119, and Pst78S84 respectively. A total of 11.42, 10.11 and 9.6 million trimmed high-quality reads were used for whole-genome assembly of Pst110S119, Pst46S119, and Pst78S84 respectively using SPAde assembler. *De novo* assembly resulted in 24300, 37795 and 24174 scaffolds (>200bp length) with 58.62, 58.33 and 55.78 Mb of assembled bases in Pst110S119, Pst46S119, and Pst78S84 respectively (Table 2, S1 Table in **S2 File**). N50 value of the assembled genome ranged from 3.9 to 7.0 kb and was highest for Pst110S119 isolate. Length of the longest scaffold ranged from 27.46 to 71.34 kb among the three pathotypes as mentioned in Table 2 and Fig 1. Our BUSCO analysis has identified 71.8% to 83.2% complete and 10.0% to 15.6% fragmented BUSCO orthologs based on conservation of 1,335 single-copy orthologs of the basidiomycota dataset [28] (S1 Fig). Furthermore, Fungal Genome Mapping Project (FGMP) pipeline [29] depicts 88.2%, 87.4%, 88.4% and 87.5% completeness of fungal conserved genes in Pst110S119, Pst46S119, Pst78S84 and DK09_11, respectively. We observed a higher genomic coverage for Pst110S119 (78.9%) and Pst46S119 (78.6%) as compared to Pst78S84 (75.2%) using *P*. *striiformis* isolate DK09_11 (genome size: 74 Mb) as a reference [67] [S1 Table in **S2 File**]. We report a lower genomic coverage compared to previously assembled Indian isolates of *P*. *striiformis* [20].

**Table 2:** Genome statistics of *Puccinia striiformis tritici* pathotypes Pst110S119, Pst46S119 and Pst78S84.

	Pst110S119	Pst46S119	Pst78S84
Number of scaffolds	24,300	37,795	24,174
Genome size (Mb)	58.62	58.33	55.78
GC content (%)	44.5	44.4	44.4
Scaffold L50/N50(kb)	2,191/7.03	4,071/3.94	2,494/6.25
Longest scaffold(kb)	71.34	27.46	54.74
Number of scaffolds > 1 kb	10,691	13,526	10,908
*de novo* repeat	12,858,610 bp (21.94%)	12,553,985 bp (21.53%)	11,620,224 bp (20.84%)
Known repeats (bp)	707544 (1.55%)	689262 (1.51%)	695891 (1.58%)
Ancestral repeats (bp)	9818624 (72.37%)	9758091 (73.68%)	8922394 (72.44%)
Lineage specific repeats (bp)	3739654 (27.56%)	3475584 (26.24%)	3388714 (27.51%)
Protein coding genes	13,744	15,163	13,758
Percentage of genome covered by genes (%)	34.5	32.6	35.2
Known homologs	11,041	11,731	11,046
Gene Ontology	7,876	8,621	7,852
Protein family	5,208	5,451	5,173

### Identification and characterization of repetitive elements in *P*. *striiformis tritici*

We applied a hierarchical approach for detecting repetitive elements including an initial detection of *de novo* repeats using RepeatModeller and RepeatClassifier followed by searching for the taxa-specific repeats using RepBase. Nearly, 21.94%, 21.53% and 20.84% *de novo* repeats were identified in Pst110S119, Pst46S119 and Pst78S84, respectively. Search for the known repeats increased the total content of repeats to 23.13%, 22.69% and 22.07% in three genomes. Both Class I (LTR, non-LTR) and Class II (DNA) elements were detected. Class I retrotransposons consisted of ~30%, whereas DNA transposons constitute ~39% in three genomes, while a larger portion of repeats 28.08% (Pst110S119), 27.74% (Pst46S119) and 26.81% (Pst78S84) remained unclassified. Copia element belonging to the LTR family constituted ~12% and Gypsy constituted ~15% of the known repeats. Lineage-specific repeats ranged from 26% to 27%, whereas the ancestral repeats constituted a major portion of 72% to 73% (Table 2, S2 Table in **S2 File**). Previous studies demonstrated wide variations in the total TE content among the *P*. *striiformis* pathotypes. TEs make about 31% to 48% of the genome in different pathotypes [16, 20, 21, 68]. The identified TEs content in the present study is less compared to the previous reports.

### Gene prediction and ortholog identification

*De novo* gene prediction using GeneMark predicted 13744, 15163, and 13758 genes covering 32.6% to 35.2% of genomic region in Pst110S119, Pst46S119, and Pst78S84 genome respectively (Fig 1A; S3 Table in **S2 File**). The largest genes ranged from 16.1 kb, 10.5 kb and 15.7 kb in Pst110S119, Pst46S119 and Pst78S84, respectively. Homology search against NCBI non-redundant protein database detected homologs for 77% - 80% (e-value 1e^-03^) of the genes in the three isolates. Gene ontology terms were assigned to 56% - 57% of the genes using Blast2GO. Protein family database search assigned 5208, 5451, and 5173 proteins domains/families to Pst110S119, Pst46S119 and Pst78S84, respectively and 1109, 1102 and 1091 enzymes classes were mapped to known enzyme pathways in Kyoto Encyclopedia of Genes and Genomes (KEGG; Ogata et al., 1999) [69] in three pathotypes (S4 Table in **S2 File**). Protein clustering assigned 75% of genes to 10962, 10923, and 10823 orthologous groups in Pst110S119, Pst46S119 and Pst78S84, respectively (Fig 1B). Across the three isolates, 9051 orthogroups were common and constitute the core gene set. https://orthovenn2.bioinfotoolkits.net/cluster/single-copy/9bac5af824af348ca60fbf79e105c60d/orthoThere were 2393 (17%), 3782 (24%), and 2501 (18%) singleton genes for Pst110S119, Pst46S119, and Pst78S84, respectively.

### Secretome and effector genes in *Pst* isolates

**Fig 2:**
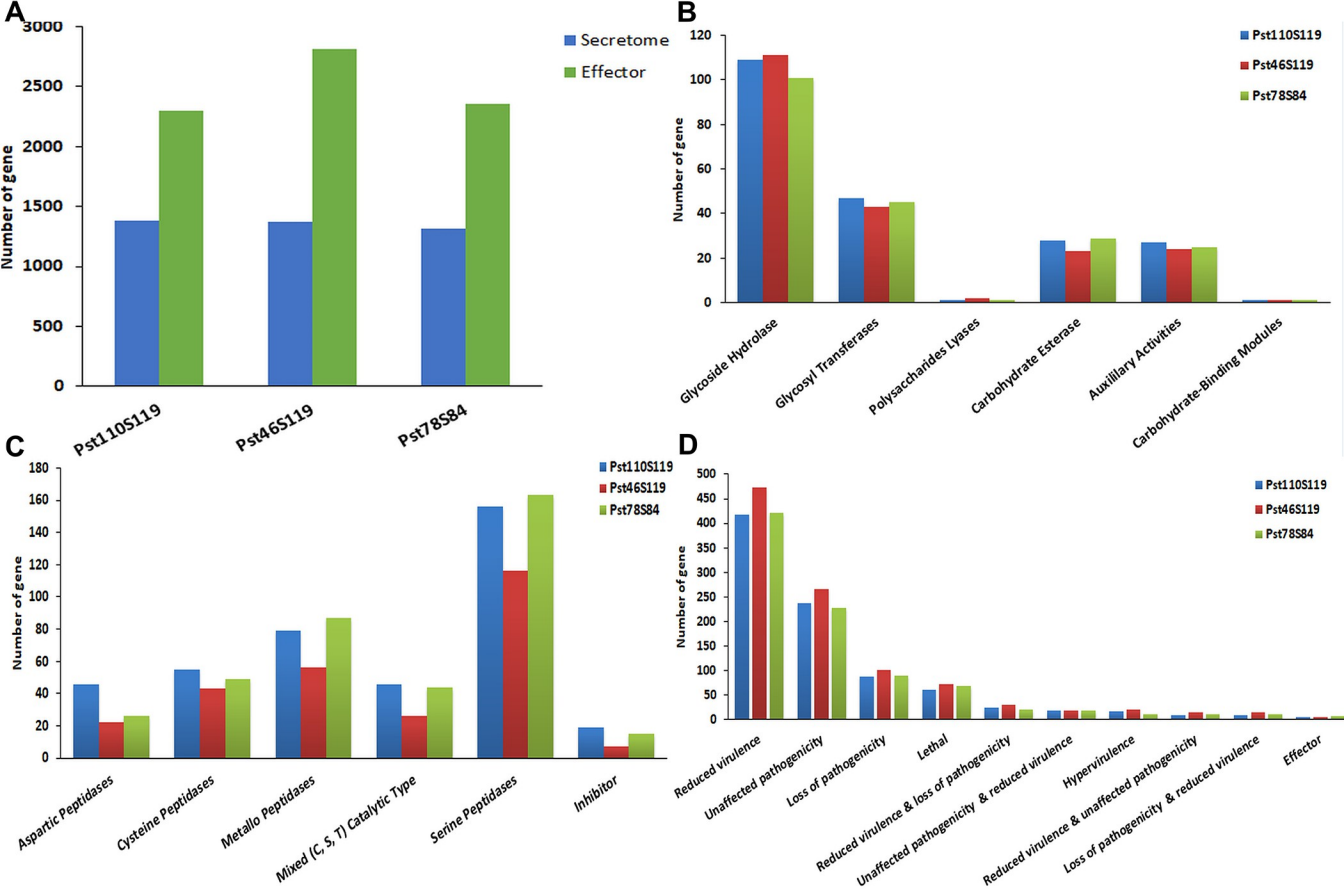
Distribution of pathogenicity gene families A) effector and secretome B) CAZyme classes C) protease classes and D) PHI-base homologs in the three Pst110S119, Pst46S119 and Pst78S84 isolates.

Secretome analysis discovered 1382 (10.05%), 1372 (9.04%) and 1312 (9.53%) extracellular proteins in Pst110S119, Pst46S119 and Pst78S84, respectively (Fig 2A; S5 Table in **S2 File**). Majority of identified secretory proteins displayed match with hypothetical proteins from other fungal species, while no homologs were detected for 80, 92, and 76 proteins in Pst110S119, Pst46S119 and Pst78S84, respectively.

Plant pathogenic fungi secrete effector proteins to facilitate infection. In this study, we have identified a total of 2302 (16.7%), 2809 (18.5%) and 2355 (17.1%) putative effectors at probability threshold score value 0.5 in Pst110S119, Pst46S119 and Pst78S84, respectively (Fig 2A; S6 Table in **S2 File**). At e-value of 1e^**-05**^ and sequence identity of more than 30%, Pst110S119 shared 2,062 and 2,053 effector orthologs with Pst46S119, and Pst78S84 respectively. Ortholog analysis using OrthoVenn categorized 4,851 effectors proteins into 1,921 clusters whereas 2,615 remained as singletons among the pathotypes. This large number of singleton effectors might be the source of variability and specificity among *Pst* pathotypes.

### Other pathogenicity determinant classes of CAZymes, proteases and PHI-base homologs

Secreted carbohydrate-active enzymes (CAZymes) are a crucial factor for fungal biological activity [70]. We detected 213, 204 and 202 genes belonging to CAZyme in Pst110S119, Pst46S119 and Pst78S84, respectively (Fig 2B, S7 Table in **S2 File**). CBM21 consisted of single gene in all three isolates. Cell wall degrading enzyme (CWDE) or plant polysaccharide degradation (PDD) enzyme plays important role in the disintegration of the plant cell wall by bacterial and fungal pathogens. PDD includes Glycoside Hydrolase (GHs), Polysaccharides Lyases (PLs) and Carbohydrate Esterase (CEs) groups of CAZYmes that are considered as a potential candidate for plant polysaccharides degradation [71]. PDD represented a significant proportion of CAZymes candidates in *Pst*, (accounting for 64–66%)). Peptidases provide an alternate carbon source for pathogens and are secreted during the infection process [72]. We have predicted 385, 258 and 362 protease genes in Pst110S119, Pst46S119, and Pst78S84 respectively using MEROPS database of protease families at an e-value of 1e^**-05**^. Five enzyme classes and one inhibitor class were identified among *Pst* proteins (Fig 2C; S8 Table in **S2 File**). Approximately 14% of putative secreted proteins belong to the protease family. Previous studies have characterized these peptidases with the ability to digest proteins under extracellular hostile environment [73]. Protease inhibitors are produced by both host and pathogen to inhibit and counter cellular proteases as part of the defense mechanism. Peptidases inhibitors belonging to class I51, I4, I87, I9, I32 were identified in isolates.

We have identified 935 (6.80%), 1072 (7.06%) and 942 (6.84%) PHI-base homologs in Pst110S119, Pst46S119, and Pst78S84 respectively (S9 Table in **S2 File**). Reduced virulence in loss of function mutants constituted the largest category with 471 to 474 genes in three isolates, followed by unaffected pathogenicity genes (228–267) and loss of pathogenicity genes (88–102). Loss of function mutants was lethal for 61–72 genes, whereas genes with increased virulence effect identified for 11–20 genes and 5–6 genes belonging to effectors in PHI-base homologs. (Fig 2D).

**Fig 3:**
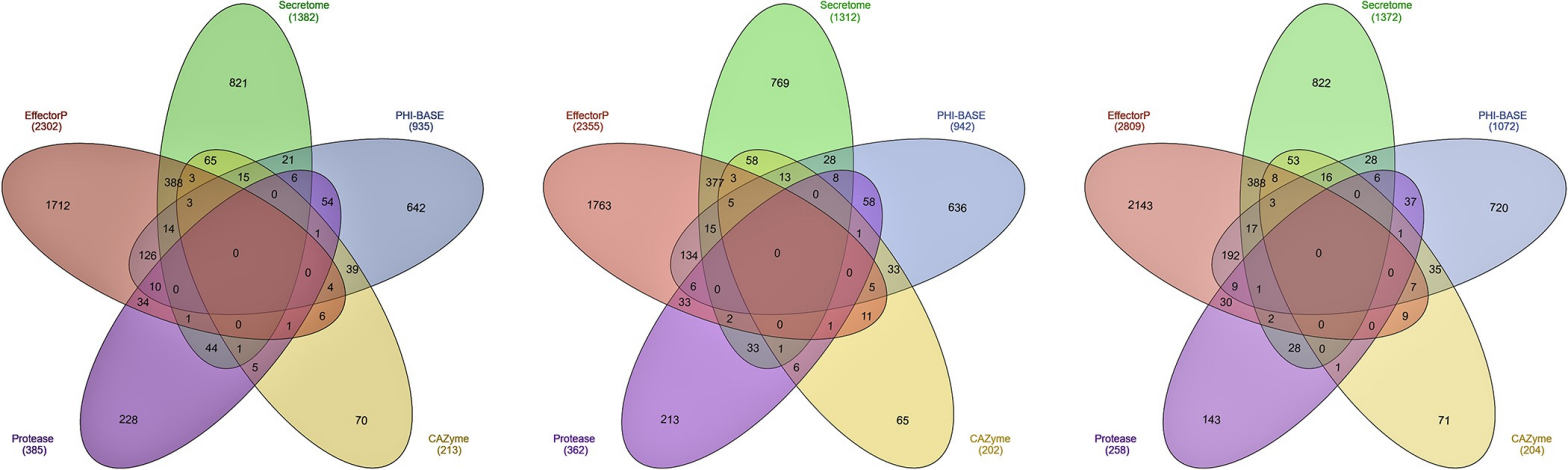
Distribution of genes among the different pathogenicity classes of secretome, effector, PHI-base homologs, proteases and CAZymes in A) Pst110S119, B) Pst78S84 and C) Pst46S119.

Comparison of gene classes from different predictions (EffectorP, Secretome, PHI-base homologs, CAZyme and *MEROPS*) gave insight into multiple roles played by pathogenicity-related genes (Fig 3). Effectors encoding genes constituted the largest cluster followed by the secretory and pathogenicity homologs from PHI-base. Approximately, 388 genes belonged to “secretory and effectors” category, 126 to 192 genes were found belonging to “effector and pathogenicity” class, followed by 21 to 28 genes belonging to “secretory and PHI-base homologs”. Pst110S119 and Pst78S84 consisted of 58 and 54 genes that belonged to the “pathogenicity and proteases” category, while only 37 genes were detected in Pst46S119. Thirty-three to thirty nines genes showed “pathogenicity and CAZyme activity”, 14–17 genes displayed “secretory, effectors and pathogenicity” activities and 3 to 5 genes showed “effector, secretory, pathogenicity and CAZyme activities” (S10 Table in **S2 File**). Description of the different pathogenicity gene classes and relationship among the three Indian pathotypes can be found in S1 File.

### Diversity of *Pst* pathotypes

Urediniospores of order *Pucciniales* consist of dikaryotic nuclei and can re-infect their primary host. The genetic variation among the nuclei can contribute towards the diversity and host adaptation and could be advantageous to the pathogen. SNP density of 3.12 ± 0.16 SNPs/kb was identified for two nuclei within a pathotype. Identified SNP density was lower than the earlier reported density of ~5 SNPs/kb (Table 3) [16, 18, 20]. To investigate the variability between the three pathotypes, inter-isolate SNPs were determined. SNPs were classified into a) homokaryotic and b) heterokaryotic SNPs as described previously [16]. SNP density of 7.51±0.19 SNPs/kb was observed, and Pst78S84 showed the highest SNP density. Approximately, 44.04±1.91% of SNPs were identical and 54.64±1.88% SNPs were variable among the pathotypes. Heterokaryotic sites were found to be more prevalent (3.73±0.19 SNPs/kb) than homokaryotic sites (0.32±0.14 SNPs/kb) among the isolates. Pst78S84 demonstrated the highest inter-isolate variability for Pst46S119 and Pst110S119. These results were in concordance with the evolutionary process depicted through the species tree. UPGMA (unweighted pair group method with arithmetic mean) tree constructed using the variant position confirmed the evolutionary closeness among the pathotypes Pst78S84 and Pst110S119 (S2 Fig).

**Table 3:** Intra and inter-isolate single nucleotide variations among *P*. *striiformis tritici* pathotypes Pst110S119, Pst46S119 and Pst78S84.

	Pst110S119		Pst46S119		Pst78S84	
Intra-pathotype SNP						
Total SNPs	188,488		186,365		163,821	
Multiallelic SNP sites	55		41		51	
SNPs/KB	3.21		3.19		2.93	
TS/TV	2.14		2.12		2.15	
Inter-pathotype SNP						
	Pst46S119	Pst78S84	Pst110S119	Pst78S84	Pst110S119	Pst46S119
Total Sites(SNPs/kb)	4,28,836 (7.32)	4,29,082 (7.32)	4,36,184 (7.48)	4,37,699 (7.50)	4,30,657 (7.72)	4,31,813 (7.74)
Identical Sites (SNPs/kb)	1,99,957 (3.41)	1,80,458 (3.08)	2,00,269 (3.43)	1,84,924 (3.17)	1,85,601 (3.33)	1,91,272 (3.43)
Homokaryotic Site (SNPs/kb)	20,641 (0.35)	20,366 (0.35)	23,974 (0.41)	24,700 (0.42)	20,832 (0.37)	20,541 (0.37)
Hetrokaryotic Sites (SNPs/kb)	2,02,234 (3.45)	2,22,717 (3.80)	207,289 (3.55)	2,20,465 (3.78)	2,20,337 (3.95)	2,13,408 (3.83)

### Gene family evolution

**Fig 4:**
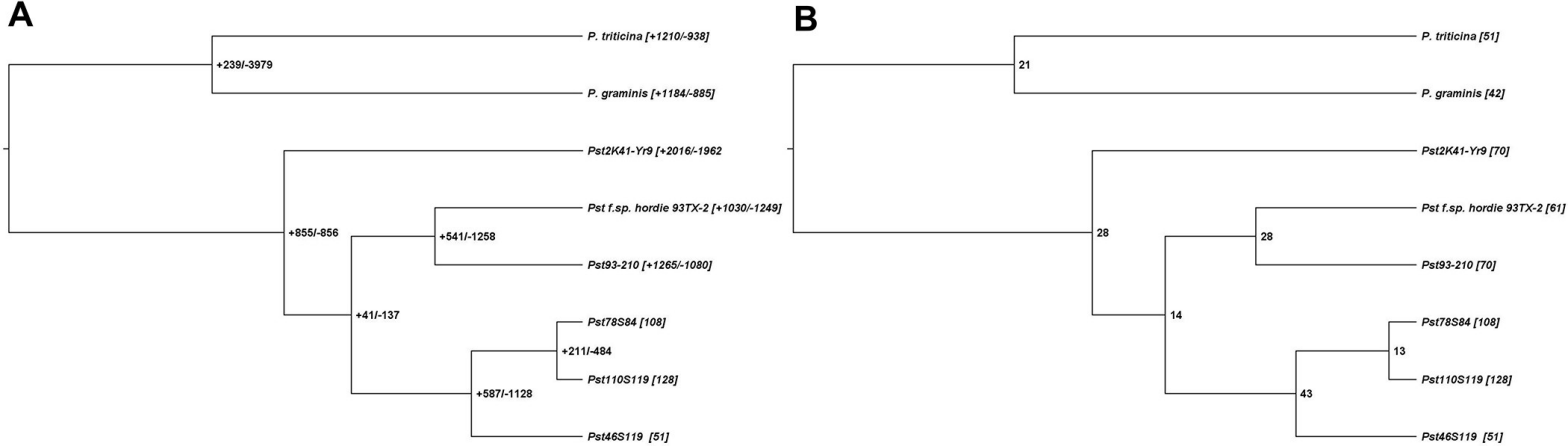
Evolution of gene families A) Gene gain and gene loss at the nodes and tips in five isolates of *Puccina striiformis tritici*, one isolate of each *P*. *striiformis* from barley, *P*. *triticina* and *P*. *graminis tritici*. Numbers in square bract shows the gene family gain (positive) and gene family loss (negative) B) Rapidly evolving gene families identified with CAFÉ among the isolates.

Evolution of gene families was examined using eight *Pst* genomes (*P*. *triticina* race 1 (BBBD), *P*. *graminis* f. sp. *tritici* (CRL 75-36-700-3), *P*. *striiformis* f. sp. *tritici* (2K41-*Yr9*), *P*. *striiformis* f. sp *tritici* (93–210), *P*. *striiformis* f. sp. *hordei* (93TX-2) and Pst110S119, Pst46S119 and Pst78S84 using CAFE. A total of 10,178 gene families were assigned using RBH blast and MCL. A uniform birth-death parameter (ƛ) of 0.0165051 was calculated, representing the rate of change of evolution in the species tree. Six hundred thirty-four families were reported to be significantly evolving (family-wide p-value ≤ 0.05), 191 were rapidly evolving (family-wide p ≤ 0.01 and viterbi p-value < = 0.01). Gene gain and gene loss estimated by CAFE for each branch of the phylogenetic tree are shown in Fig 4A. Extensive gene family loss was observed at internodes compared to gene family gain, indicating shedding of genes during the speciation or divergence event. More gain of gene families was observed at terminal nodes between *P*. *triticina* and *P*. *graminis* compared to internode, attributed to their early divergence. Terminal nodes or leaves displayed more gain in the gene families compared to internode in the tree. Among the Indian pathotypes, Pst110S119 showed a significant loss in gene families, while Pst46S119 gathered more gene families. Higher number of rapidly evolving gene families were identified between the Indian pathotypes. Terminal branch with the maximum number of rapidly evolving gene families was the one with Pst110S119 and Pst78S84 with 128 and 108 genes, respectively (Fig 4B). The higher rate of rapidly evolving gene families provided strong evidence for the rapid adaptation of *P*. *striiformis* to the changing environment.

More contraction was observed for secretome and effector proteins compared to PHI-base homologs among three pathotypes. A higher rate of gene family expansion was observed in Pst110S119 and Pst78S84 compared to Pst46S119. The contraction of these groups indicated rapid adaptation by pathogens to changing environments. Pathotypes Pst110S119 and Pst78S84 showed rapidly evolving genes classes under selection (S11 Table in S2 File; S3 Fig). Description of the gene classes under rapid selection is presented in S1 File.

### Genes under positive selection

**Fig 5:**
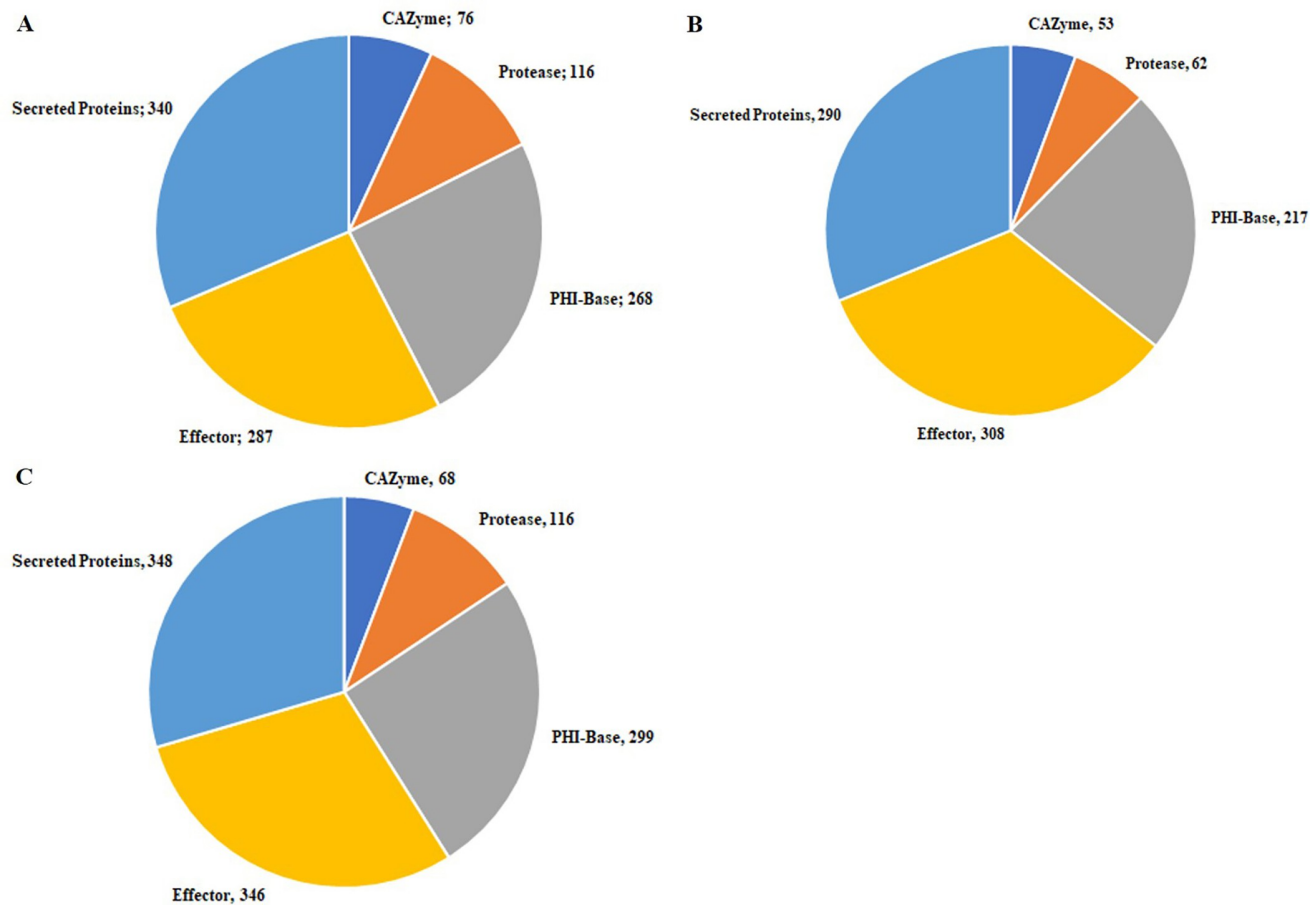
Fast evolving positively selected genes of CAZyme, protease, PHI-base homologs, secreted protein and effector protein in three *Pst* pathotypes A) Pst110S119, B) Pst46S119 and C) Pst78S84.

A total of 5611 (Pst110S119), 4797 (Pst46S119) and 5646 (Pst78S84) complete gene sequences with at least 4 homologs from the six *P*. *striiformis* genomes (*P*. *striiformis* f. sp. *tritici* (2K41-*Yr9*), *P*. *striiformis* f. sp *tritici* (93–210), *P*. *striiformis* f. sp. *hordei* (93TX-2) and Pst110S119, Pst46S119 and Pst78S84) were used for detection of evolutionary selection pressure acting on genes. Sequences with p<0.001 were considered significant and were further used in the study. A total of 3118, 2644 and 3264 genes were found to be under the positive selection in Pst110S119, Pst46S119 and Pst78S84, respectively (Fig 5; S12 Table in **S2 File**). Recently evolved pathotypes Pst110S119 and Pst78S84 showed higher number of genes under positive selection. Among these, carbohydrate-active enzymes consisted of 76, 53, and 68 genes that were under positive selection in Pst110S119, Pst46S119, and Pst78S84 respectively. Glycoside Hydrolase formed the largest group followed by Glycosyl Transferases, Auxiliary Activities, Esterase Carbohydrate, and Polysaccharide Lyase (S4 Fig). In Pst78S84, a single carbohydrate-binding module was found to be rapidly evolving. Among the proteases, 116 genes displayed rapid selection in Pst78S84 and Pst110S119 whereas, in Pst46S119, sixty-two genes were under selective forces (Fig 5). Serine peptidase formed the largest group in rapidly evolving genes followed by “Mixed (C, S, T) Catalytic Type” proteases, Metallopeptidases, Cysteine peptidases, Aspartic peptidases and inhibitors. Pst46S119 displayed fewer evolving classes compared to the other two isolates. S5 Fig displays the distribution of the fast-evolving protease classes. PHI-base homologs consisted of 268, 217 and 299, secretome consisted of 340, 290 and 348 and effectors consisted of 287, 308 and 346 genes under positive selection in Pst110S119, Pst46S119 and Pst78S84, respectively (Fig 5).

### Association of transposable elements (TEs) with pathogenicity genes classes

**Fig 6:**
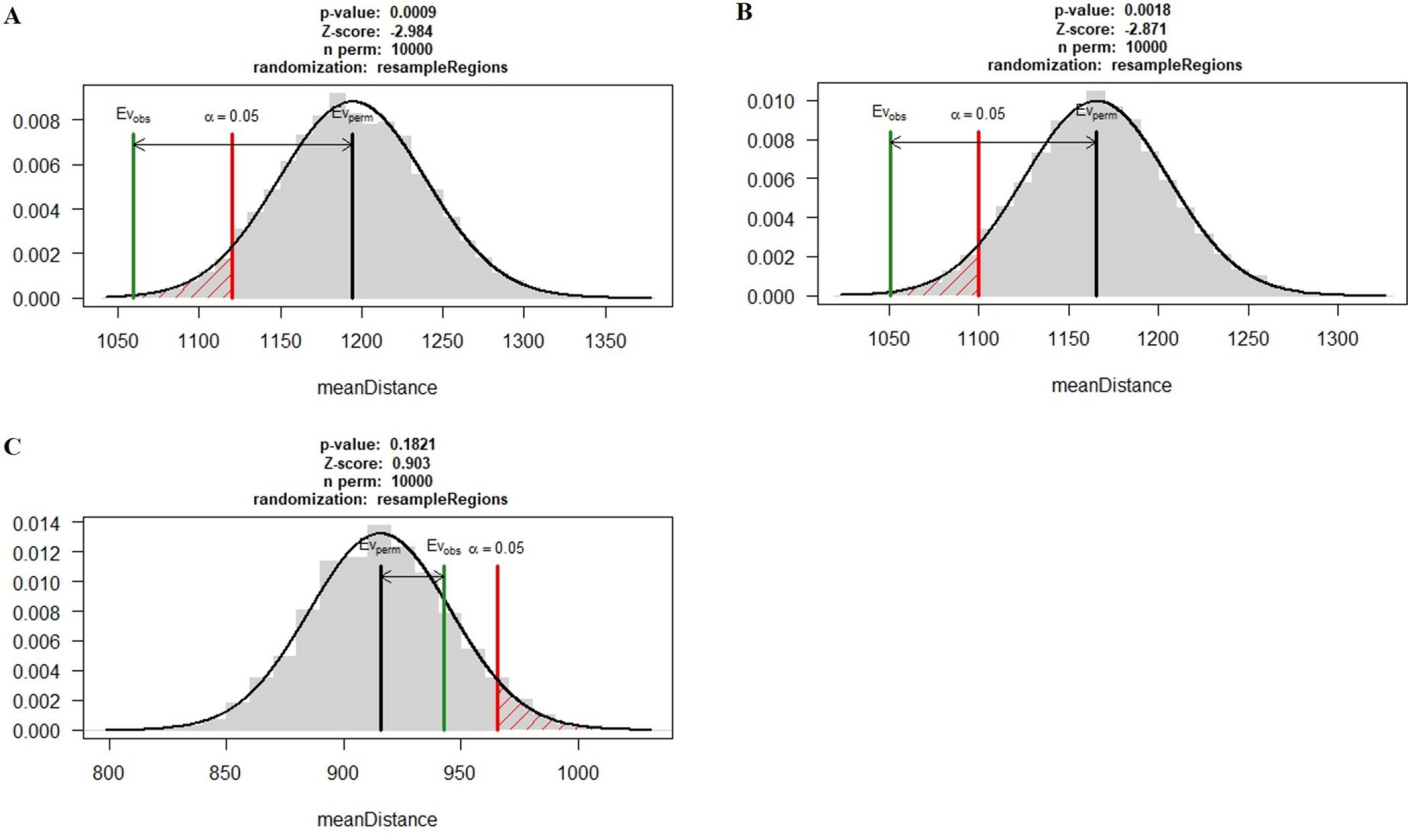
Association between the genomic repeats and effector gene in pathotypes. Black bar represents the mean distance of random sampling from the repeats, red bar indicates the significance limit and green bar represent the mean of observed distance for the effector genes from the repeats. A) Pst110S119, B) Pst78S84, and C) Pst46S119.

Mean distance of the pathogenicity gene classes of CAZyme, proteases, genes involved in pathogen-host interaction, candidate effectors, and secretome proteins of three *Pst* pathotypes were compared to genomic repeats. We sampled 10,000 random permutations using regioneR [44], and the mean distance of the selected pathogenicity genes was compared to the position of genomic repeats. P-values were calculated for each gene class by sampling the distribution of mean values (S13 Table in **S2 File**). P-values demonstrated that genes belonging to the effector category were more closely associated with TEs in Pst110S119 and Pst78S48 compared to other gene classes (p-value <0.001). No significant association of gene classes and TEs were detected for Pst46S119 (Fig 6). Effector genes showed a significant p-value (0.00089) and Z-score (-2.98) for Pst110S119, and p-value (0.00179) and Z-score (-2.87) for Pst78S84 (S14 Table in **S2 File**). A negative Z-score for the effectors in Pst110S119 and Pst78S84 indicated that these were more closely linked to the genomic repeat elements (S6 Fig). The observed mean distance for protease, PHI-base homologs and secretome was higher than the mean for random sampling. For CAZyme p-value was higher than the significant limit of 0.05. “Pathogen host interaction” and secretome genes showed a significant p-value in Pst46S119 but the observed mean distance was more than the mean of random sampling (S6 Fig).

### Phylogenetic analysis

**Fig 7:**
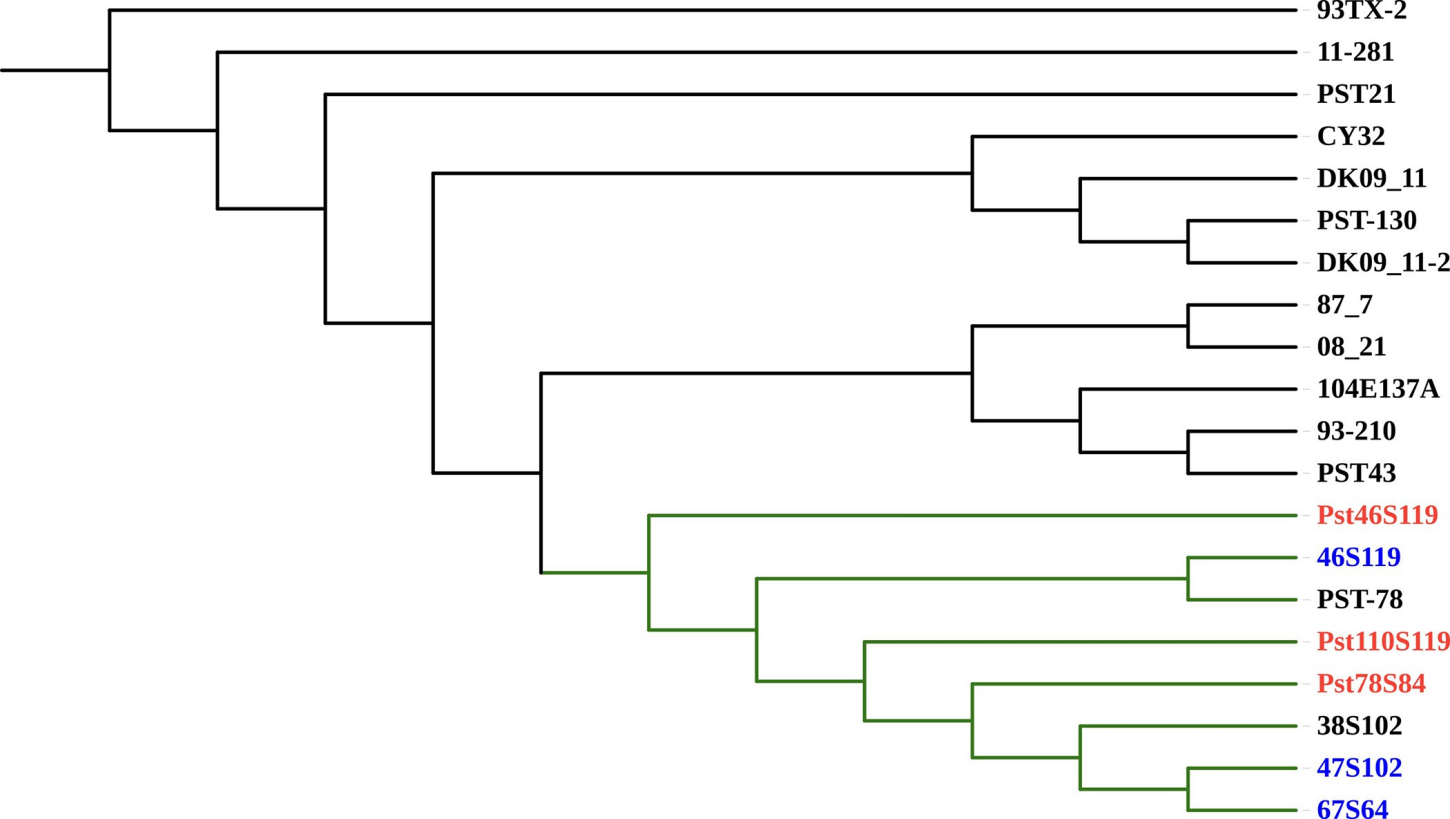
Phylogenetic tree based on the orthologous gene alignment of conserved BUSCO genes in 20 *Pst* pathotypes: USA (11–281, 93–210, 93TX-2, PST-78, PST-130, PST21, PST43), UK (08/21, 87/7), Denmark (two assemblies for DK09_11), Australia (104E137A), China (CY32), India (38S102, 46S119, 47S119, 67S64) and Pst110S119, Pst46S119, Pst78S84.

BUSCO based conserved gene analysis of 17 published *Pst* genomes and three presently sequenced genomes showed coverage ranging from 33% to 90% in *Pst* pathotypes (S7 Fig). BUSCO based phylogeny grouped the six Indian *Pst* pathotypes (three Pst genome from Kiran et. al., 2017) into a single phylogenetic clade (Fig 7). A set of 1698 single-copy orthologous (SCO) identified using OrthoFinder was used to build species-specific phylogeny of *Puccinia* pathotypes (*P*. *triticina* race 1 (BBBD), *P*. *graminis* f. sp. *tritici* (CRL 75-36-700-3), *P*. *striiformis* f. sp. *tritici* (2K41-*Yr9*), *P*. *striiformis* f. sp. *tritici* (93–210), *P*. *striiformis* f. sp. *hordei* (93TX-2) and Pst110S119, Pst46S119 and Pst78S84). All six *P*. *striiformis* isolates were grouped into one clade, whereas *P*. *triticina* and *P*. *graminis* formed the outgroup **(**S8 Fig**)**. Indian pathotypes Pst110S119, Pst46S119, and Pst78S84 grouped into a single clade signified their early divergence. A substitution rate of 0.04 was estimated per substitution site. Estimated substitution rate calculated using codeml program of paml package [74] was ~0.17x10^-8^ changes per site per time unit for the species tree. To understand the evolutionary relationship among the previously sequenced pathotypes (46S119, 47S102 and 67S64) from India (Kiran et al. 2017) and the *Pst* pathotypes assembled (Pst110S119, Pst46S119 and Pst78S84) in the present study along with reference strain (DK09_11), pairwise sequence identity between the isolates was calculated using mummer [75]. Two distinct groups were identified: group I consisted of DK09_11, 46S119, 47S102 and 67S64 (Kiran et al. 2017) and group II consisted of Pst110S119, Pst78S84, and Pst46S119 (S9 Fig). The sequence identity among the isolates ranges from 97% to 99%, where DK09_11 and 46S119 were most similar. Lower identity value for the Pst78S84 from other pathotypes demonstrates its recent evolution. QUAST based sequence comparison of 46S119 sequenced by Kiran *et al*. (2017) and Pst46S119 in the present study displayed a match of 74% of genomic fraction between the two and covered ~52 Mb of aligned sequence and a mismatch ratio of 0.83% per 100kb.

Local genomic regions evolve differentially from the whole genome and may provide a differential evolutionary pattern for species evolution. In the whole-genome alignment of seven *Puccinia* genomes (2K41-*Yr9*, Pst110S119, Pst46S119, Pst78S84, 46S119, 47S102 and 67S64) with reference genome DK09_11, forty-one different phylogenetic patterns were detected. S10 Fig showed the neighbor-joining tree generated from the local phylogeny cactus (SAGUARO element). Topology of the tree was different from the pairwise sequence identity tree and species tree. Nearly, 114,467 local fragments with variable length ranging from 1bp to 4.2 Mb were identified. S11 Fig displays the breakpoint between the genomic segments with reference DK09_11. Segment assigned to the most common cactus or phylogenetic region shown in dark grey, were shared between the different pathotypes and may have the differential evolutionary advantage for the pathogen.

### Identification of strain-specific genes and design of genome-specific markers

Gene prediction was conducted in 17 available *Puccina striiformis* genomes in NCBI. Ortholog analysis using OrthoFinder detected 6 (12 genes), 8 (23 genes) and 1 (2 genes) species-specific orthogroup in Pst110S119, Pst46S119 and Pst78S84, respectively, whereas 408 (Pst110S119), 838 (Pst46S119) and 441 (Pst78S84) genes remain unassigned as singletons and were specific to the particular pathotypes. Sequence-based clustering grouped 1724 pathotype specific genes ranging from 39 amino acids to 851 amino acids into 1712 clusters with 36, 54 and 34 effector genes in three genomes. These effectors may be the potential novel effector candidates and formed two major clades in the phylogenetic tree (S12 Fig). There were 175, 288 and 155 unannotated genes in three isolates. Gene specific markers were developed using kmer based approach (S15 Table in **S2 File**). Genome specific region based on k-mer approach consisted of 6.1, 7.2 and 4.9 Mb in Pst110S119, Pst46S119 and Pst78S84, respectively (S13 Fig).

### Validation of the primers

**Fig 8:**
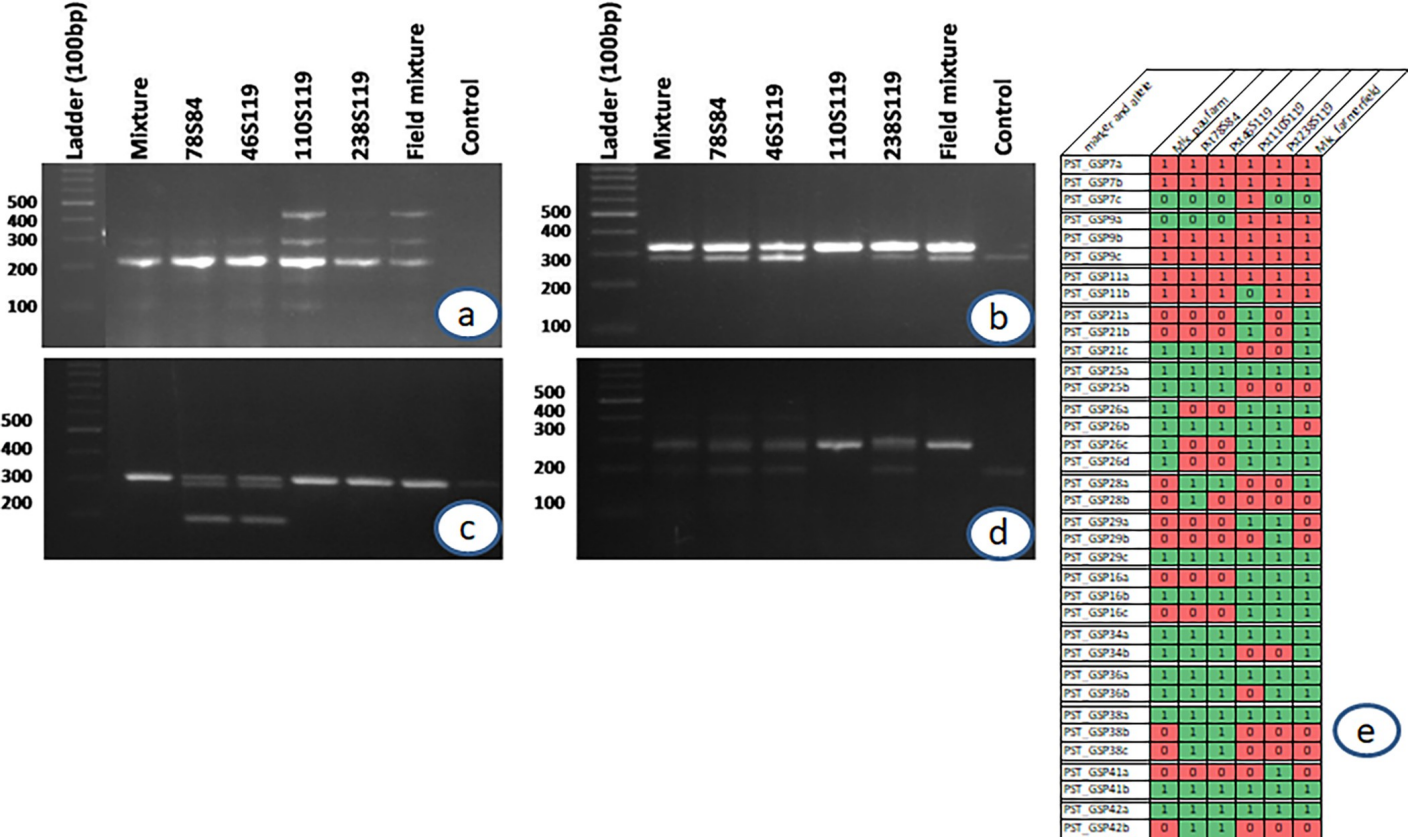
PCR amplification profile of *Pst* pathotypes for gene specific markers PST_GSP9 (a), PST_GSP11 (b), PST_GSP38 (c), PST_GSP25 (d) and barcoding of *Pst* pathotypes and field samples based on 15 gene specific markers.

Out of the 45 genome specific markers 14 (PST_GSP 7, 9, 11, 16, 21, 25, 26, 28, 29, 34, 36, 38, 41, 42) showed polymorphism (Fig 8A and 8B). The marker PST_GSP7, PST_GSP9, PST_GSP11, PST_GSP21 and PST_GSP36 were able to differentiate the pathotype 110S119 from the other two. The marker PST_GSP28 can be used for distinguishing 78S84 and 46S119, but it showed null allele in pathotype 110S119 (Fig 8B). The pathotypes 110S119 and 238S119 could be differentiated by using the marker PST_GSP29 (Fig 8B). Overall, a combination of these markers can be used to assess the prevalence of different pathotypes in the field samples.

## Discussion

We assembled 58.62 Mb, 58.33 Mb and 55.78 Mb of the genome for Pst110S119, Pst46S119 and Pst78S84, respectively representing ~78% of the genome in comparison to reference pathotype strain DK09_11. Nearly, 70 to 80% of complete BUSCO genes and ~87% of FCGs (Fungal Conserved Genes) were identified in the assembly. Previous studies reported nearly about 15,000 to 25,000 genes in *P*. *striiformis* [16, 18, 20, 21]. A potential reason for the lower gene count may be the fragmented assembly of the three isolates in the present study. We characterized the different pathogenicity genes classes (CAZyme, proteases, PHI-base homologs, secretome, and effectors) and performed phylogenetic analysis to gain insight into the evolution of these recently evolved strains/isolates.

We performed the estimation of the association of pathogenicity gene classes to their nearest genomic repeats. The results displayed a significant association of effector genes in Pst110S119 (p-value <0.0009, Z-score: -2.984) and Pst78S84 (p-value <0.0018, Z-score: -2.871). No significant association was observed for CAZyme, protease, secretome and PHI-base homologs with the repeats. Significant association of the effectors supports their rapid evolution in pathotypes.

Phylogenetic analysis of 20 *P*. *striiformis* genomes, 17 previously published genomes and three genomes sequenced in the present study grouped the six Indian *Pst* pathotypes into a single clade, displaying their recent evolution. Singh *et al*., [13], while studying the virulence profile of *Pst* isolates from the sub-mountainous region of Punjab, reported stronger clustering of 46S119 and 110S119 compared to 78S84 [13]. Phylogenetic study was in concordance with the pathogen emergence [4–6, 76]. Similar topology was observed with whole-genome alignments using SAGUARO. A higher number of species-specific genes and rapidly evolving genes among the three isolates demonstrated the involvement of diverse gene classes in the evolutionary process.

The developed gene-specific molecular markers were validated on the reference strains used in the study along with the recently evolved and most aggressive pathotype 238S119 and stripe rust inoculum collected from farmer’s field in Roopnagar district of Punjab which has been identified as hot spot area for stripe rust occurrence (10,11) and from PAU, Ludhiana Research Farm (mixture). Out of the 45 gene-specific markers designed, 14 showed polymorphism and were able to differentiate the reference strains well from each other. Such kind of marker system can help in the quick identification of pathotypes and can aid the surveillance system well to generate beforehand information which will further aid in designing better management strategies temporally and spatially. A broader set of markers have been developed from the reported genome sequences of three *Pst* pathotypes which will be validated on farmers’ field samples from across the Punjab State.

Duan *et*. *al*., (2010) reported higher genetic diversity and lower linkage disequilibrium using AFLP markers among the *Pst* strains from the Gansu region of China, compared to the isolates from France, hypothesizing the reproductive mode compared to clonal mode [77]. Himalayan ranges with the highest genotypic diversity, recombination population and high sexual reproductions rates have been considered as the potential center of *Pst* origin [78]. Adaptation to the higher temperature in southern France supports co-evolution and local adaptation of the southern *Pst* isolates versus northern strains [79].

### Heterozygosity of dikaryotic nuclei

In dikaryotic fungi, intra-nuclear polymorphism between the two nuclei of the strain contributes to its rapid evolution and host adaptation. A cause for this polymorphism may be the spontaneous mutations occurring in one of the nuclei, thus resulting in high heterozygosity. Cuomo *et al*., [19] reported high heterozygosity of the *Pst* compared to *P*. *triticina* and *P*. *graminis*. Several studies reported the genetic variations within the dikaryotic nuclei and in different populations. We observed reduced intra-genome heterozygosity in the three Indian isolates in comparison to previous studies (Table 2) [16, 18, 20]. Inter-isolate SNP density was found to be similar to previous studies, but with lesser heterokaryotic sites.

### Pathogenicity genes of *P*. *striiformis tritici*

In the present study, we reported a large array of genes involved in pathogenicity (effectors, secretome, proteolytic enzymes, plant cell wall degrading enzymes and pathogenicity homologs from PHI-base) in sequenced *Pst* pathotypes. Pathogenic fungi have been shown to consist of a higher number of CAZyme. Moolhuijzen *et al* [80] reported that biotrophic fungi consist of fewer CAZyme classes compared to necrotrophic and hemibiotrophic fungi. Among the secreted protein families of cell wall degrading enzymes (polysaccharide deacetylase), proteases (Serine carboxypeptidase: PF00450.21, Aspartic protease: PF00026.22), superoxide dismutase (PF00080.19), glycoside hydrolase family (Glyco_hydro), Glyoxal oxidase (PF07250.10) and Cutinase (PF01083.21) were found to be highly enriched in numbers. We detected 3 to 4 proteins belonging to the CFEM domain (PF05730.10), having a role in appressorium development and pathogenicity of the isolates [81]. The distribution of the predicted effectors was similar to the previous study of *P*. *striiformis* [22]. Myb/SANT-like DNA-binding domain (PF12776.6) containing effectors reported in *P*. *striiformis* translocate into the plant nucleus and help in reprogramming the transcription host immune response. Serine and Aspartic peptidases constitute the largest protease group.

### Genes undergoing positive selection

Evolution of genes is marked by the presence of non-synonymous mutations in the genome resulting in the change of underlying amino acids in the codon. The ratio of non-synonymous to synonymous substitutions (Ks/Ks), is widely used for the estimation of positive and purifying selection at the amino-acid site [53]. We identified a larger portion of the genes in Pst110S119 (22%), Pst46S119 (17%) and Pst78S84 (23%) which were under positive selection. Protease coding genes constitute the highest percentage (~50%) under positive selection in each pathotype showing the active role played by proteases followed by the pathogenicity homologs from PHI-base, CAZyme, secretome and effector genes. Majority of these genes under positive selection do not fall under any classified category and belong to diverse cellular pathways. These results support the fact that diverse gene classes were under positive selection in the pathogen. Majority of the unclassified genes belonged to hypothetical protein from the *Puccinia* genus, making it difficult to annotate their potential function. More genes in Pst110S119 and Pst78S84 were detected under positive selection compared to Pst46S119, supporting their recent emergence and diversification. We can conclude that Pst46S119 is undergoing purifying selection as also reported by [20].

### Evolution of gene families in *Pst*

The emergence of new genes with novel functions is related to the adaptive evolution of species [82]. Ethyl methanesulfonate (EMS) induced mutagenesis proved to be effective in the identification of avirulence genes in *Pst* pathogens [83]. Phylogenetic tree of gain/loss of gene families displayed topology similar to the species tree. Gene family loss was observed at all the branches (Fig 5A). The highest gene loss was observed between 93–210 and 93TX-2 contributed to their adaptation to different hosts (wheat and barley). Further, high gene gain/loss in 2K41-*Yr9* could be attributed to its early emergence. More rapidly evolving genes families and higher gene gain/loss observed in Pst110S119 and Pst78S84 can be linked to their recent emergence. Speciation has been shown with uneven gene losses at different branches in the tree of life. In plants and fungi, gene loss followed the polyploidy events. The reductive evolution based on gene loss has been demonstrated as a major force affecting evolution in all organisms [84]. Pietro *et*. *el*., [85] showed the extensive gene losses of genes belonging to primary and secondary metabolism, carbohydrate-active enzymes, and transporters in powdery mildew. In the present study different gene families displayed a diversified pattern of gene gain or loss in the three isolates. CAZyme demonstrated a higher rate of loss of gene families in Pst46S119, whereas the observed rate of gain in gene families was nearly the same in three isolates. Gene family loss in proteases also seems to be more predominant in Pst46S119 compared to gene family gain in Pst110S119 and Pst78S84. PHI-Base homologs displayed more gene loss than gene gain. Secretome and effector genes displayed a widespread gene loss and gain of new and rapidly evolving gene families. Gene loss constitutes a measure of adaptive evolution in the rapidly changing host environment.

For the successful management of stripe rust or for deployment of stripe rust resistance genes in a particular area, knowledge about the virulence pattern of the pathogen population existing in that area and their evolutionary history is required. Differential based race identification method in use for the monitoring the changes in virulence pattern of the pathogen requires longer time, trained experts and moreover they are not preferred to track the rust pathogen pathotypes at the global level by the scientists due to limited genetic differentiation. The solution for this problem is to develop a marker-based system as it is very quick and sensitive or to develop a pathotype specific platform based on whole genome sequencing of the existing *Pst* pathotypes to better understand the variable nature of the pathogen and in turn to develop effective breeding strategies. The markers developed in the present study will play a significant role in the early detection of pathotypes in the Punjab region and will help in the timely deployment of management strategies by the extension workers.

## Conclusions

Plant pathogen evolves at a higher rate in a continuously changing environment, overriding genetic resistance in crops like wheat. Novel approaches for managing these infections are necessary, which can be facilitated by the whole genome sequencing of the whole spectrum of pathogen variability of a particular region. In this study, we evaluated the pathogenicity genes and developed specific markers for strain identification using the whole genome sequence of three recently evolved *P*. *striiformis* pathotypes from the Northwestern Plains Zone of India. Gene annotation, identification of effector genes and secretome, phylogenetic analysis w.r.t. global pathotypes helped in understanding the evolutionary processes responsible for pathogen variability. The markers would be valuable for early detection and tracking of strains prior to a disease outbreak, allowing management techniques to be implemented more quickly.

## Supporting information

S1 File (DOCX)

S2 File (XLSX)

S1 Raw images (PDF)

S1 Fig (JPG)

S2 Fig (JPEG)

S3 Fig (JPG)

S4 Fig (JPG)

S5 Fig (JPG)

S6 Fig (JPG)

S7 Fig (JPG)

S8 Fig (JPG)

S9 Fig (JPG)

S10 Fig (JPG)

S11 Fig (JPG)

S12 Fig (JPG)

S13 Fig (JPG)
