# Supplementary material for: Whole genome resequencing and comparative genome analysis of three Puccinia striiformis f. sp. tritici pathotypes prevalent in India
Source: PLoS One. 2022 Nov 3;17(11):e0261697. doi: 10.1371/journal.pone.0261697 (PMC9632834; doi:10.1371/journal.pone.0261697)
Supplement: S1 File — (DOCX) [file pone.0261697.s001.docx]

Supplementary Information:

**Supplementary material for gene class comparison of three Indian *Pst* isolates.**

### Secretome

### Of the total secretome, we found 1,202 orthologous clusters among the three isolates (614 single copy ortholog clusters and 588 clusters with at least genes from two isolates). Here, singletons constituted a larger portion of secreted proteins (Pst110S119: 21%, Pst78S84: 22% and Pst46S119: 28%). Fifty percent of the proteins among isolates were part of 643 core clusters. Pst110S119 and Pst78S84 displayed highest number of 868 orthogroup clusters (2,411 genes) followed by Pst110S119 and Pst46S119 with 825 orthogroup clusters (1,327 genes) and Pst46S119 and Pst78S84 with 778 clusters (2,231 genes). Three hundred ninety distinct protein families/domains were identified accounting for ~27 % of the secretome in the three isolates. Pst110S119, Pst46S119 and Pst78S84 consisted of 381, 377 and 356 distinct domains.

### Effector

### Predicted effectors varied in length from 39 to 392 amino acids between the isolates with an average size ranging from 145 to 147 amino acid. Protein domain search assigned 803 distinct domains to 590, 823 and 640 effector proteins in Pst110S119, Pst46S119 and Pst78S84, respectively. Blast search revealed homologs for 2,122 (Pst110S119), 2,595 (Pst46S119) and 2,184 (Pst78S84) effector proteins. There were 180 (Pst110S119), 214 (Pst46S119) and 171 (Pst78S84) effector proteins without any known homolog. Extracellular proteins constitute 409, 419 and 403 effector genes in Pst110S119, Pst46S119 and Pst78S84, respectively.

### One-to-many orthologous relationships was identified between the effector genes. Nearly 565 (Pst46S119) and 600 (Pst78S84) genes displayed 100% identity with Pst110S119 genes. Pst46S119 and Pst78S84 consisted of 2,410 orthologs, of which 506 were 100% identical.

### CAZymes

Among the CAZymes identified using dbCAN-CAZymes classification pipeline, GH consisted of ~50% of the classified enzymes in three isolates. CBM21 consisted of single gene in all three isolates. Cell wall degrading enzyme (CWDE) or plant polysaccharide degradation (PDD) enzyme plays important role in the disintegration of the plant cell wall by bacterial and fungal pathogens. PDD represented a significant proportion of CAZymes candidates in *Pst*, accounting for 64-66 percent. Glycoside Hydrolase family 18 (GH18), a class of fungal chitinases, was found highly enriched with the highest number (11 to 13) of genes in all three pathotypes. The Auxiliary Activities (AAs) class consist of ligninolytic enzyme families, which are not directly involved in the degradation of carbohydrates but cooperate with classical polysaccharide depolymerase (<http://www.cazy.org/Auxiliary-Activities.html>). AAs consisted of 22-25 genes in each genome. Orthogroup assignment using Orthovenn clustered 576 (93%) proteins from the three isolates into 197 clusters, of which 155 were single copy orthologs, while 13, 19 and 11 CAZyme proteins remained as singleton in Pst110S119, Pst46S119 and Pst78S84, respectively.

### Proteases

### Pst110S119 consisted of the highest number of secreted peptidases followed by Pst78S84 and Pst46S119. Serine peptidases were found to be highly enriched followed by Metallo-peptidases and Aspartic peptidases in three isolates. Among the serine peptidases, genes belonging to family S9, S10 and S33 were more abundant (Table S8). Inhibitor class I51 known to work under the acidic pH and inhibiting S10 peptidases family members were abundant in Pst110S119 and Pst78S84. Inhibitor classes I9 and I32 were not detected in Pst46S119. Orthovenn clustered 914 peptidases into 345 clusters, whereas 37, 22 and 34 proteases were located only in Pst110S119, Pst46S119 and Pst78S84, respectively.

**Gene family evolution of Proteases, CAZyme, effector, Phi-base homologs and secretome**

We also investigated the evolutionary pattern of pathogenicity-related gene families, i.e. CAZyme, proteases, effector, Phi-base homologs and secretome to gain insight into gene evolution. CAZyme genes of three isolates were grouped into 128 families using the MCL program. Three singleton gene families were removed from the analysis. In Pst110S119, Pst46S119, and Pst78S84, CAFE predicted the expansion of 7, 5 and 5 gene families and contraction of 5, 13 and 9 gene families, respectively (**Table S11; Figure S3**). Two rapidly evolving CAZyme families were identified in Pst78S84.In Pst110S119, gene expansion comprised of members from Glycoside Hydrolase (GH5_41, GH5_5, GH2, GH105), Auxiliary Activities (AA1_3), Carbohydrate Esterase (CE4) and Glycosyl Transferases (GT15), whereas some members of the Glycoside Hydrolase (GH7, GH47, GH47) and Carbohydrate Esterases (CE5, CE4) displayed gene contraction. In isolate Pst46S119, members of Glycoside Hydrolase (GH18, GH47, GH13_22) and Auxiliary Activities (AA5_1) displayed expansion, whereas Glycoside Hydrolase (GH32, GH71, GH17), Carbohydrate Esterases (CE8, CE10, CE10, CE10, CE10), Auxiliary Activities (AA1, AA1_3, AA9) and Glycosyl Transferases (GT4, GT33) displayed gene family contraction. In Pst78S84, Glycoside Hydrolase (GH5_9, GH47, GH71, GH81, GT50) showed expansion while Glycoside Hydrolase families (GH5_41, GH79, GH10, GH131, GH76, GH43_24, GH26, GH20) and Auxiliary Activities family AA9 showed contraction.

Among the Proteases, CAFÉ predicts a total of 290 gene families for *Pst* pathotypes. Expansion of 18, 2 and 16 families and contraction of 8, 101 and 14 gene families were observed for Pst110S119, Pst46S119 and Pst78S84, respectively (**Table S11**). Pst78S84 displayed two fast-evolving genes families. In Pst110S119, all six groups of proteases showed expansion i.e., Serine Peptidases (5), Aspartic Peptidases (2), Inhibitor (3), Metallo Peptidases (3), Mixed Catalytic Type (3), Cysteine Peptidases (2), whereas some members of these families also displayed contraction Metallo Peptidases (2), Serine Peptidases (2), Cysteine Peptidases (2), Aspartic Peptidases (2). In Pst46S119, two families of Serine Peptidases and Mixed (C, S, T) Catalytic type exhibited expansion while the members of Serine Peptidases (35), Metallo Peptidases (24), Aspartic Peptidases (7), Mixed (C, S, T) Catalytic type (17), Inhibitor (8) and Cysteine Peptidases (10) displayed contraction. In Pst78S84, members of Metallo Peptidases (5), Serine Peptidases (9) and Mixed (C, S, T) Catalytic Type (2) were detected undergoing expansion, while some classes of Serine Peptidases (4), Aspartic Peptidases (2), Mixed (C, S, T) Catalytic Type (1), Cysteine Peptidases (2), Metallo Peptidases (4), Inhibitor (1) displayed contraction. More contraction was observed for secretome and effector proteins compared to PHI-base homologs among three isolates. A higher rate of gene family expansion was observed in Pst110S119 and Pst78S84 compared to Pst46S119. The contraction of these groups indicated rapid adaptation by pathogens to changing environment. Pathotypes Pst110S119 and Pst78S84 showed rapidly evolving genes classes under selection (**Table S11; Figure S3**).
