# Supplementary material for: Whole genome resequencing and comparative genome analysis of three Puccinia striiformis f. sp. tritici pathotypes prevalent in India
Source: PLoS One. 2022 Nov 3;17(11):e0261697. doi: 10.1371/journal.pone.0261697 (PMC9632834; doi:10.1371/journal.pone.0261697)
Supplement: S1 Raw images — (PDF) [file pone.0261697.s003.pdf]

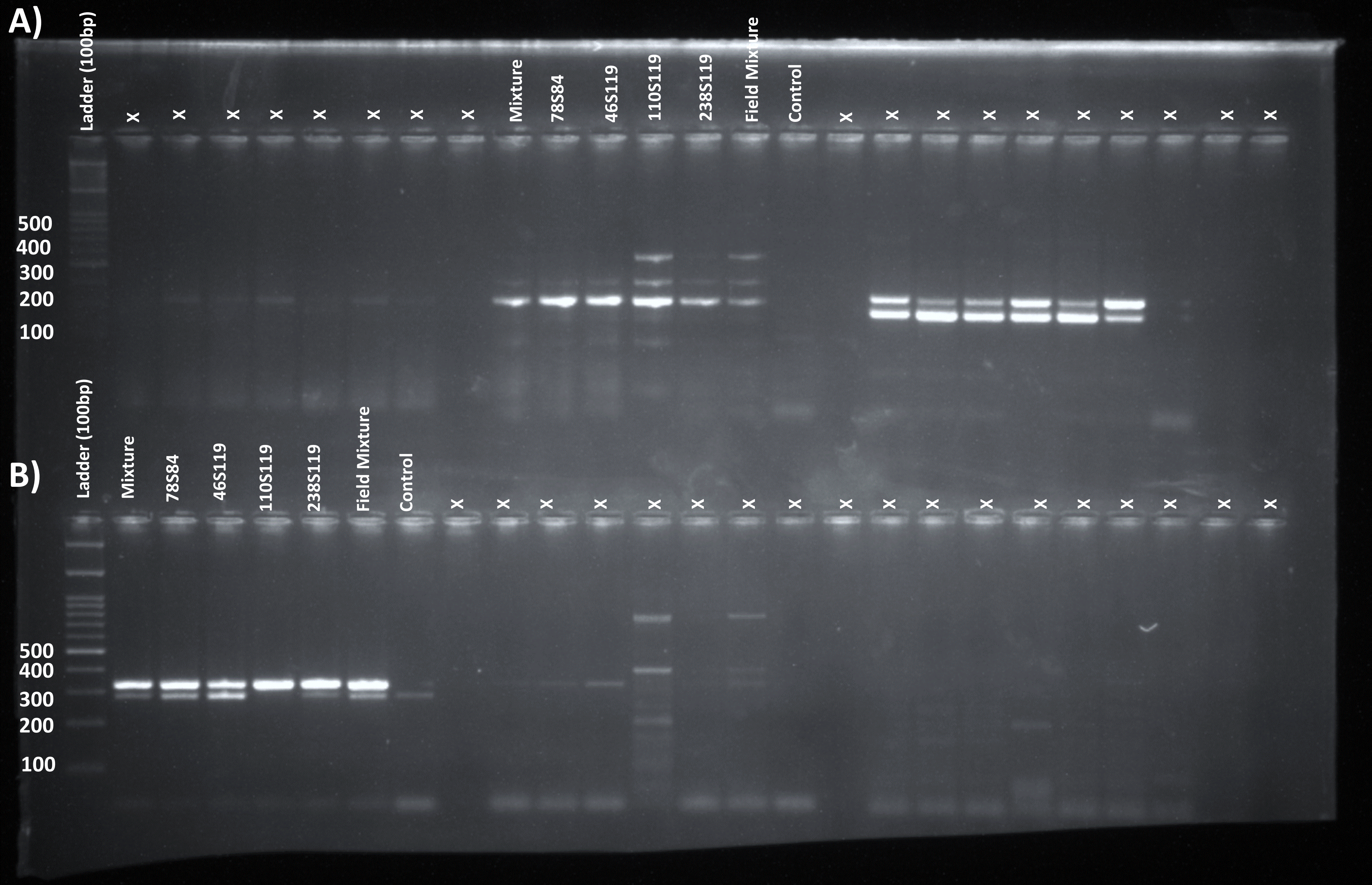
A)
Ladder (100bp)
X
X
X
X
X
X
X
X
Mixture
78S84
46S119
110S119
238S119
Field Mixture
Control
X
X
X
X
X
X
X
X
X
X
500
400
300
200
100
B)
Ladder (100bp)
Mixture
78S84
46S119
110S119
238S119
Field Mixture
Control
X
X
X
X
X
X
X
X
X
X
X
X
X
X
X
X
X
X
500
400
300
200
100

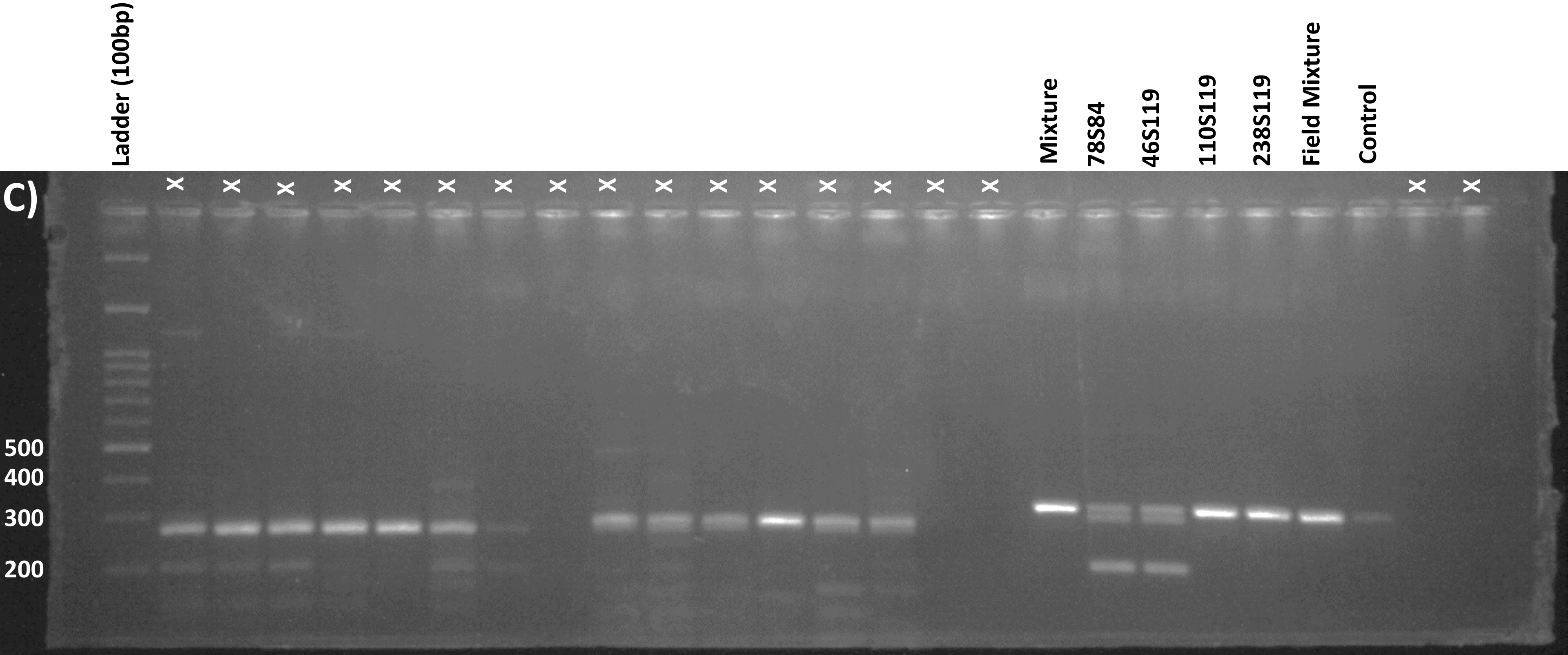

C)
Ladder (100bp)
Mixture
78S84
46S119
110S119
238S119
Field Mixture
Control
500
400
300
200

D)

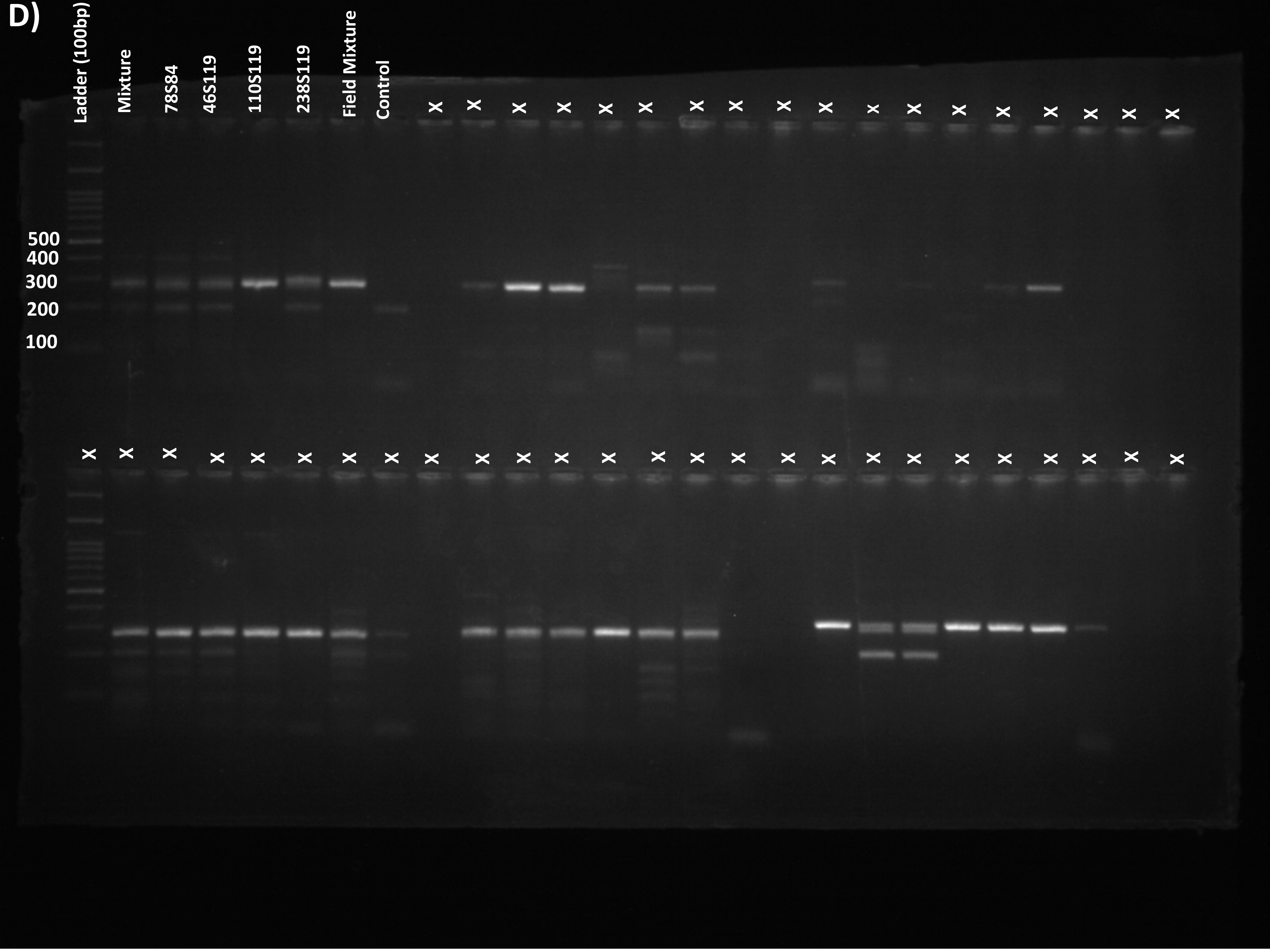
Ladder (100bp)
Mixture
78S84
46S119
110S119
238S119
Field Mixture
Control
500
400
300
200
100
