# Supplementary figures and images for: Whole genome resequencing and comparative genome analysis of three Puccinia striiformis f. sp. tritici pathotypes prevalent in India
Source: PLoS One. 2022 Nov 3;17(11):e0261697. doi: 10.1371/journal.pone.0261697 (PMC9632834; doi:10.1371/journal.pone.0261697)

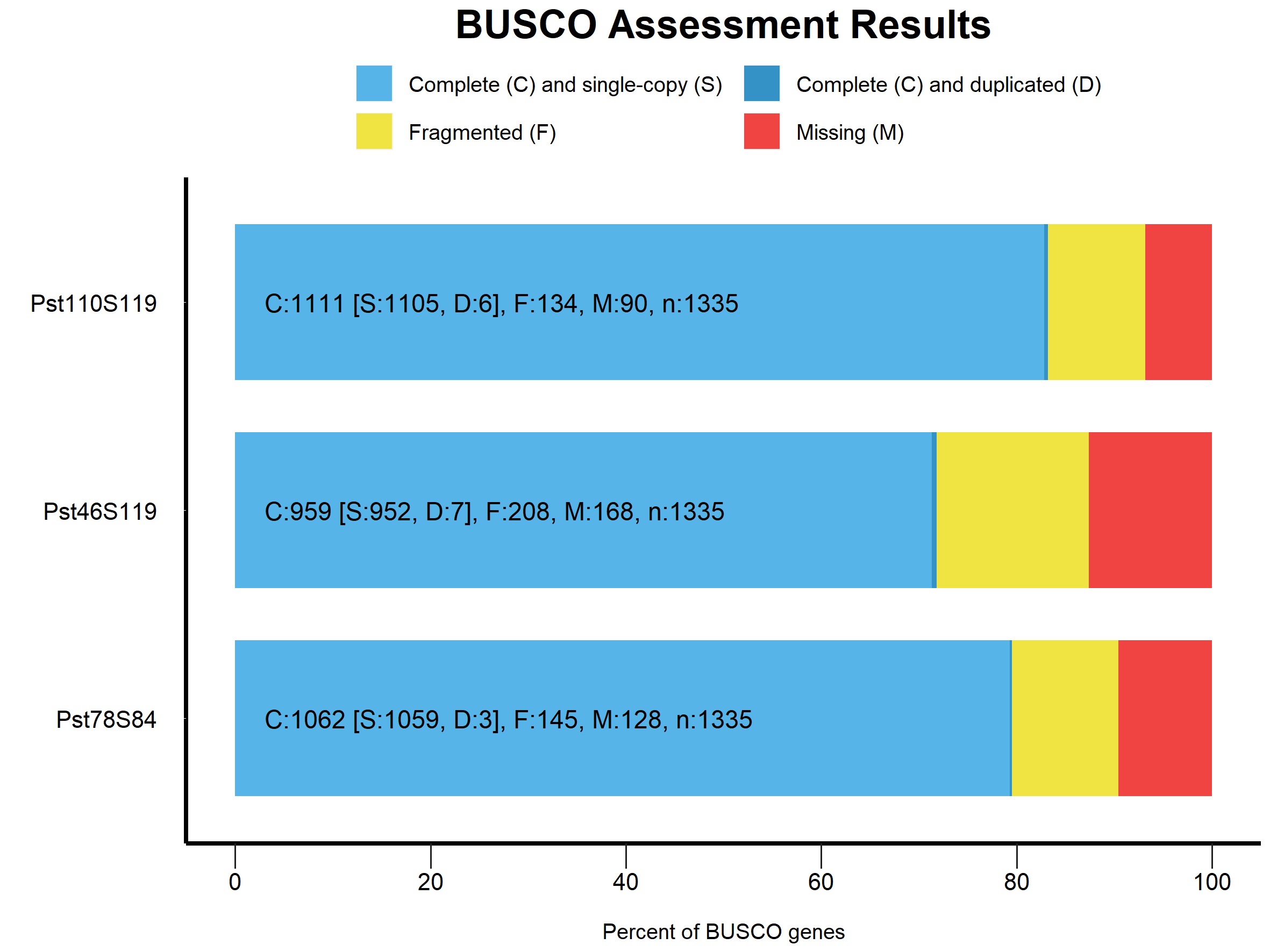

Supplement: S1 Fig — (JPG) [file pone.0261697.s004.jpg]

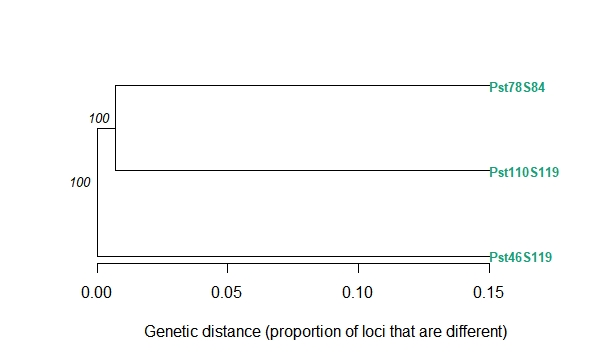

Supplement: S2 Fig — (JPEG) [file pone.0261697.s005.jpeg]

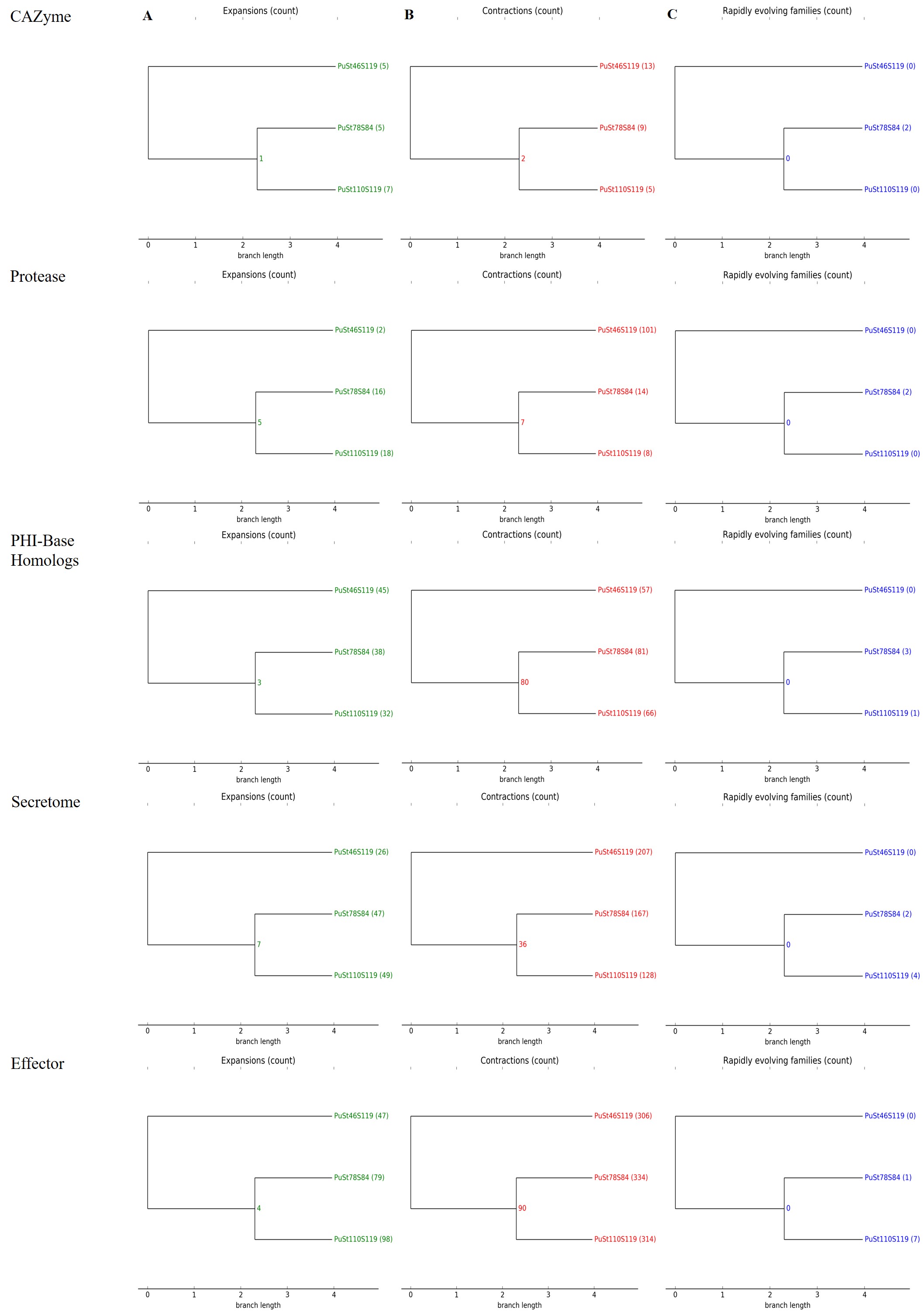

Supplement: S3 Fig — (JPG) [file pone.0261697.s006.jpg]

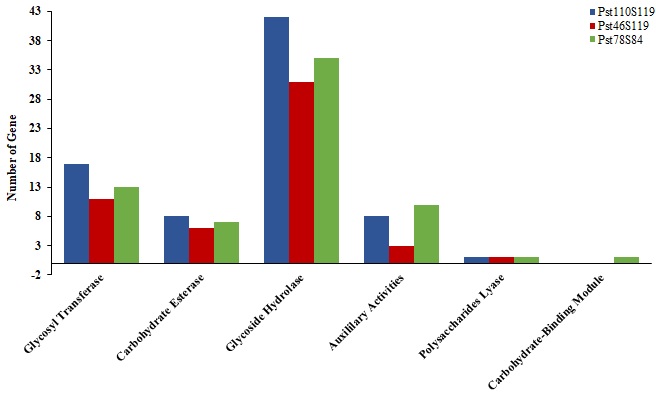

Supplement: S4 Fig — (JPG) [file pone.0261697.s007.jpg]

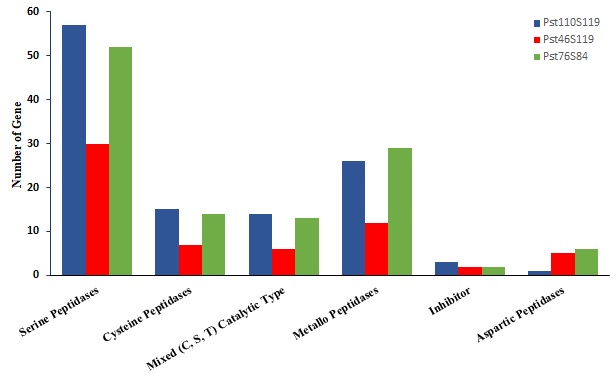

Supplement: S5 Fig — (JPG) [file pone.0261697.s008.jpg]

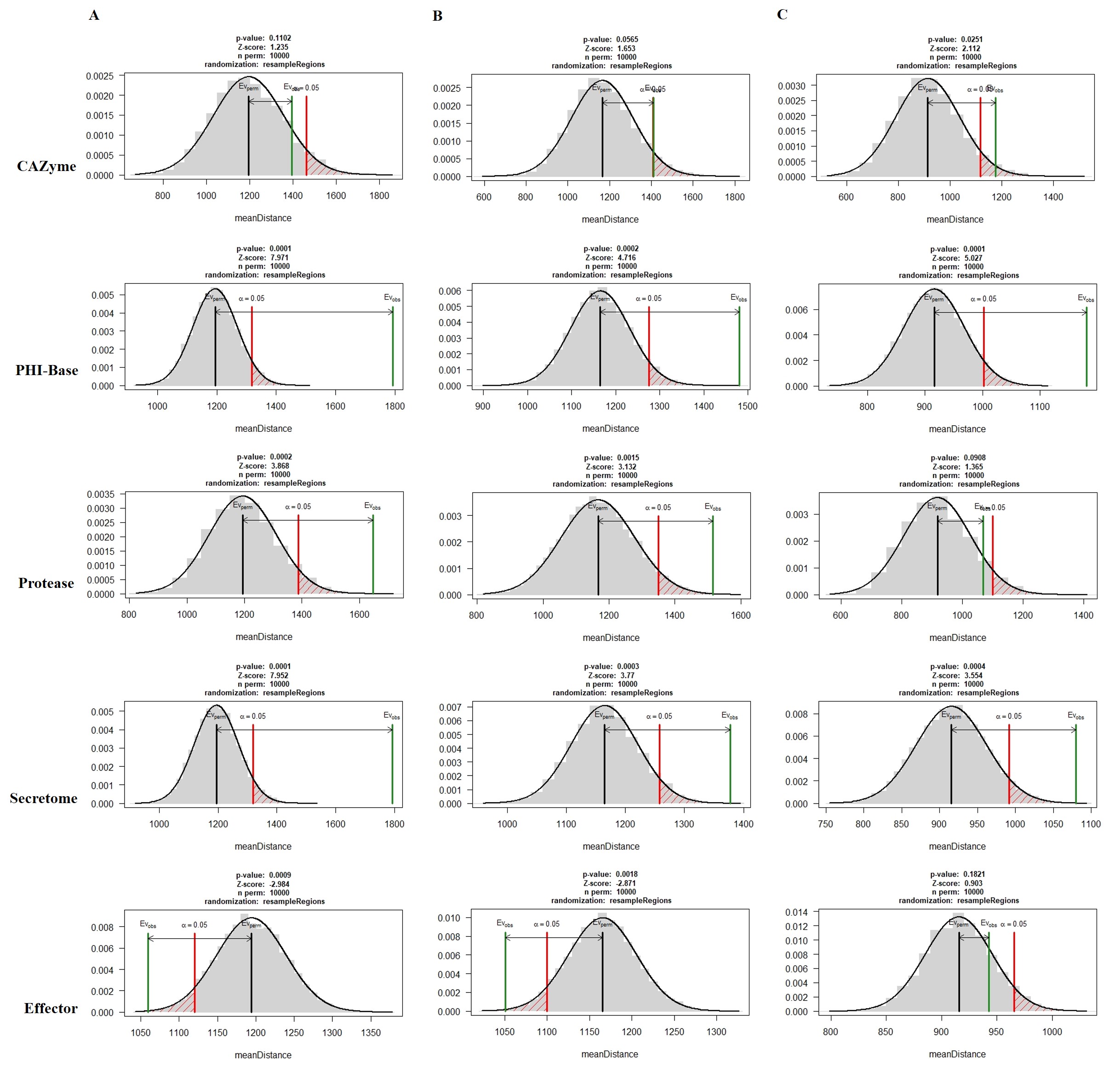

Supplement: S6 Fig — (JPG) [file pone.0261697.s009.jpg]

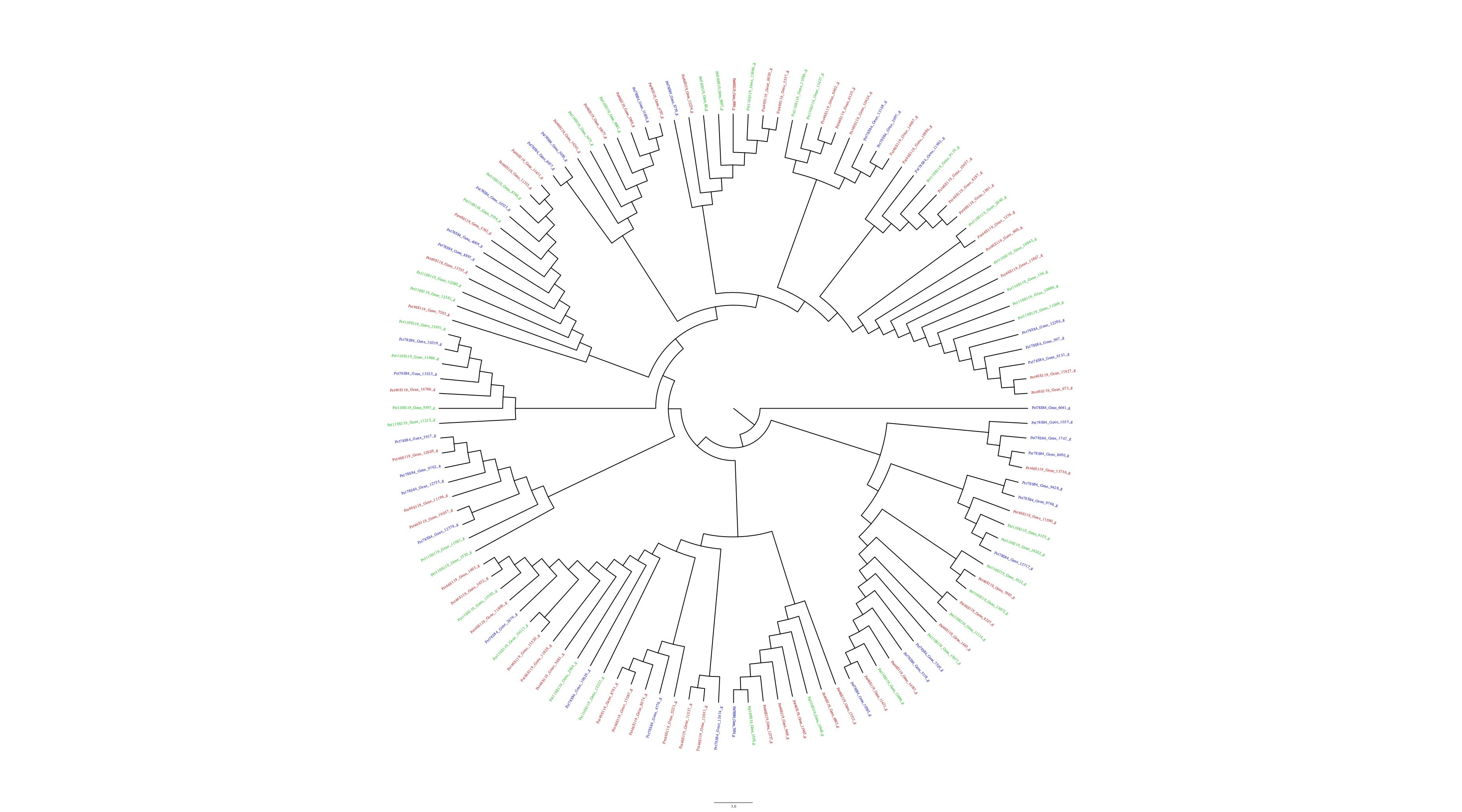

Supplement: S7 Fig — (JPG) [file pone.0261697.s010.jpg]

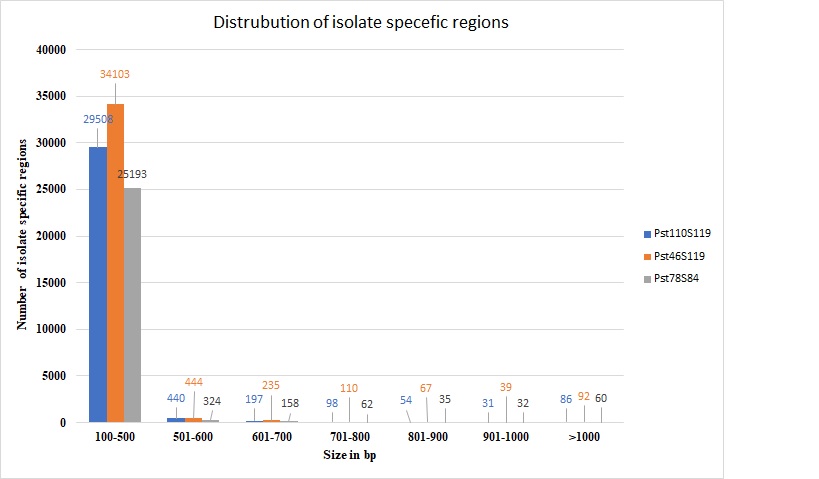

Supplement: S8 Fig — (JPG) [file pone.0261697.s011.jpg]

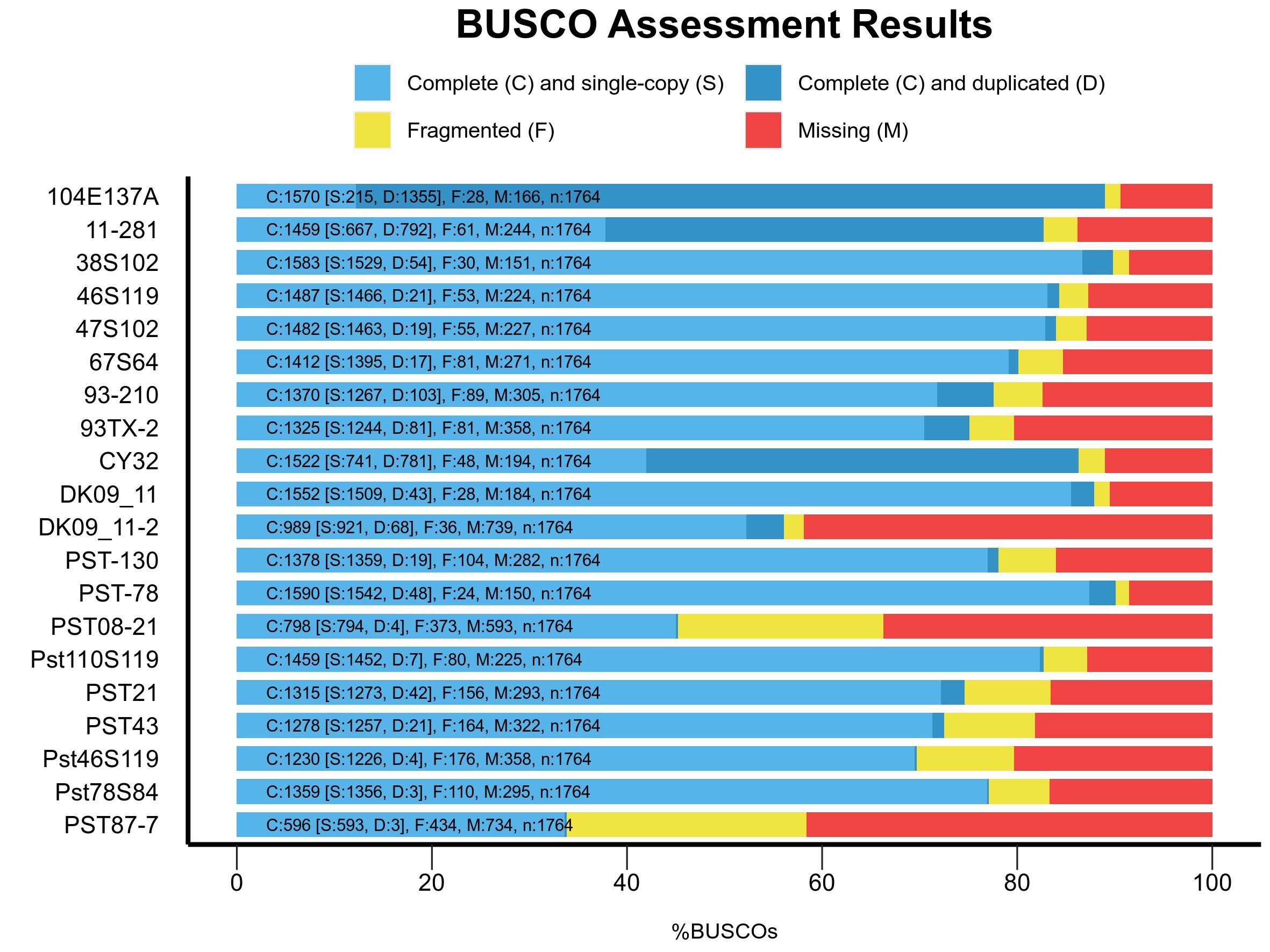

Supplement: S9 Fig — (JPG) [file pone.0261697.s012.jpg]

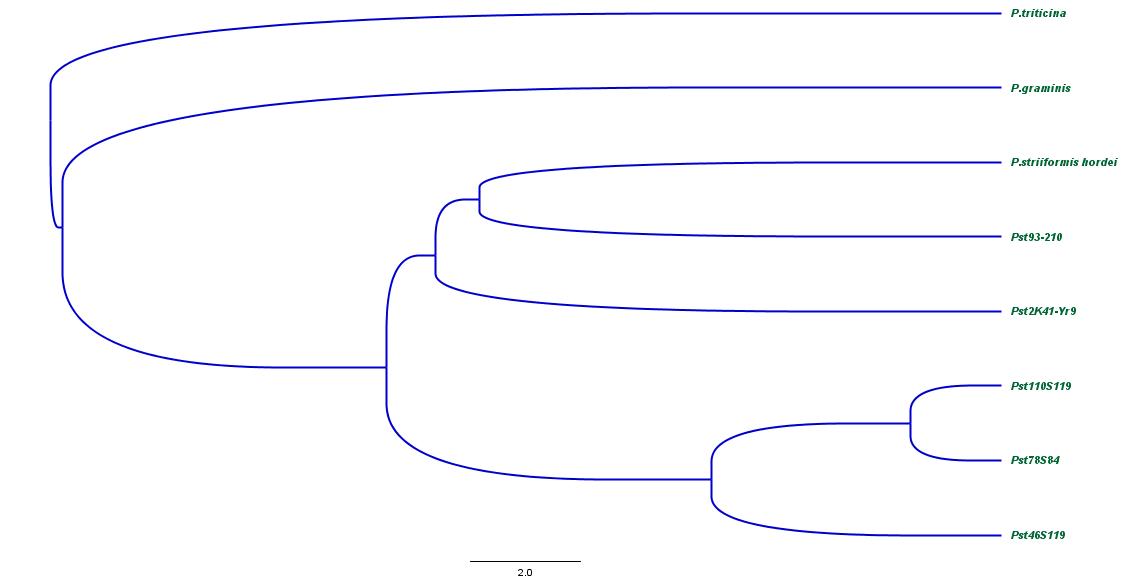

Supplement: S10 Fig — (JPG) [file pone.0261697.s013.jpg]

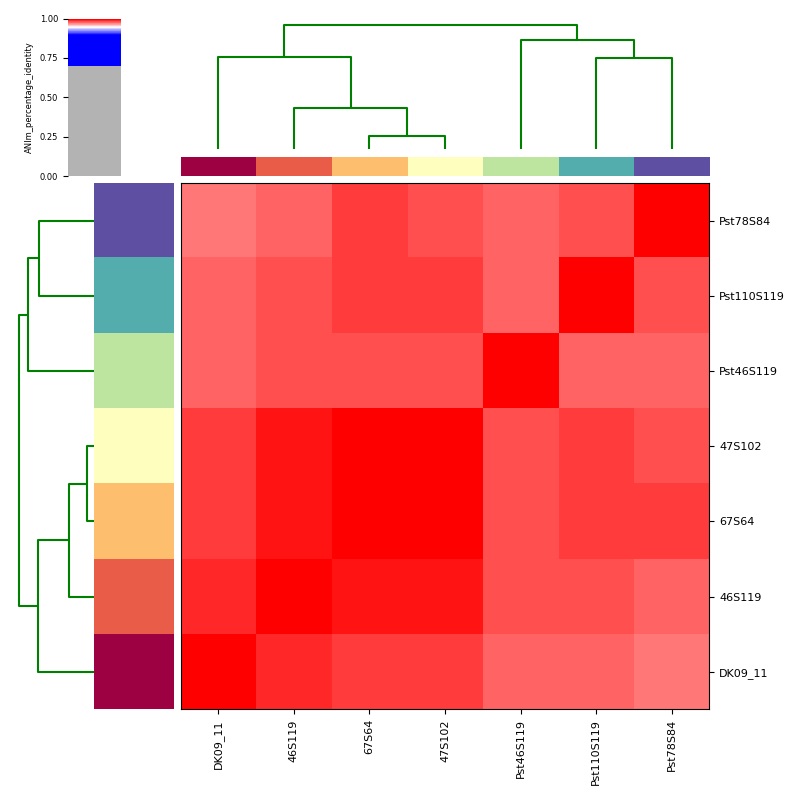

Supplement: S11 Fig — (JPG) [file pone.0261697.s014.jpg]

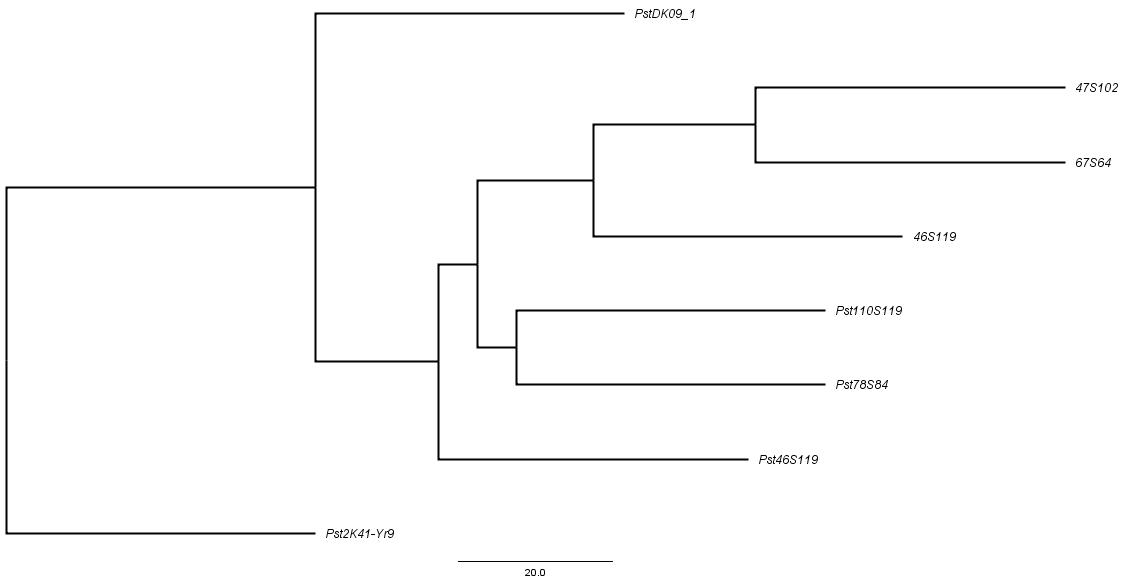

Supplement: S12 Fig — (JPG) [file pone.0261697.s015.jpg]

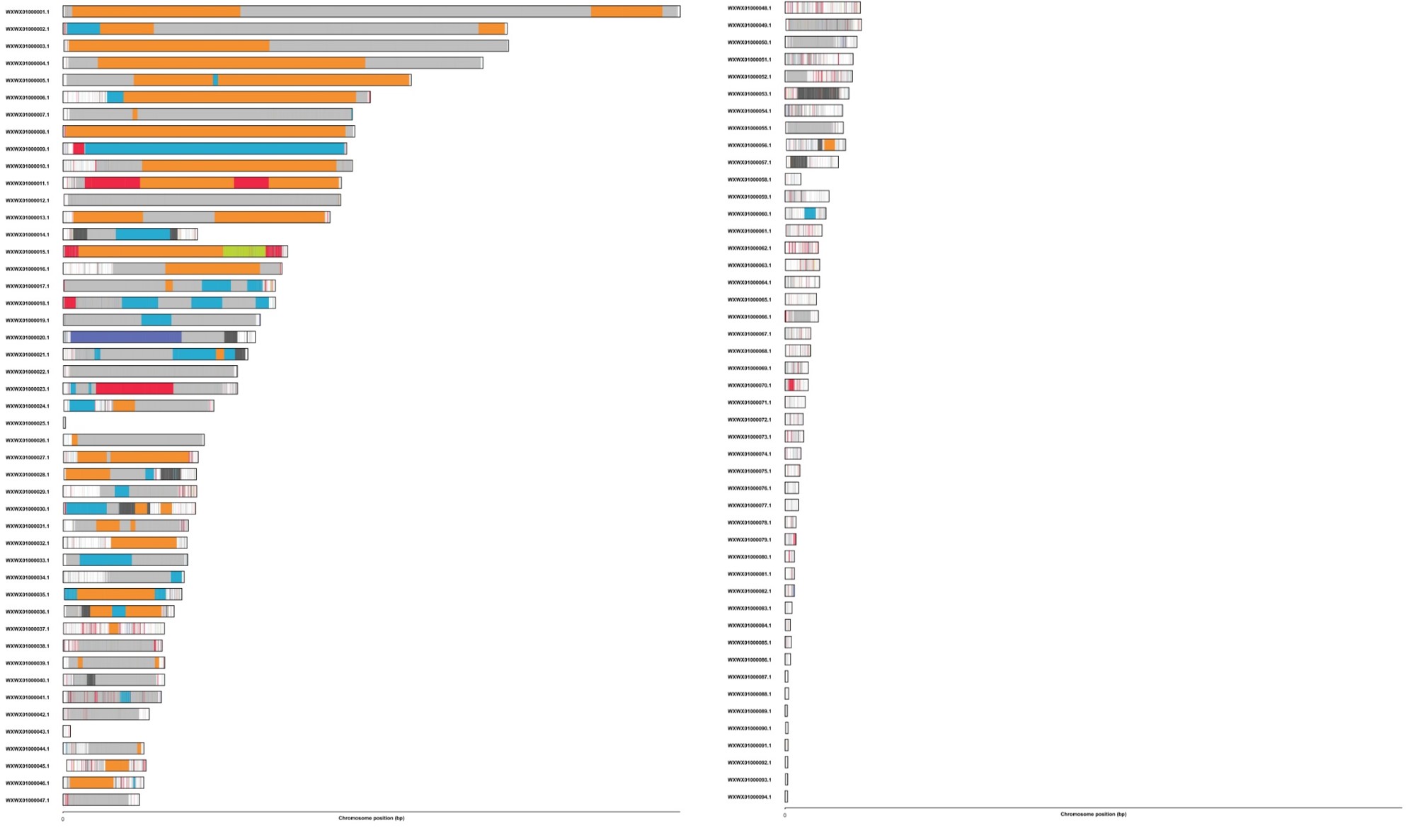

Supplement: S13 Fig — (JPG) [file pone.0261697.s016.jpg]
